# Barriers and facilitators of cardiac rehabilitation focusing on underserved groups: a mixed-methods systematic review

**DOI:** 10.1136/bmjopen-2026-119214

**Published:** 2026-10-29

**Authors:** Hanan A Humoud, Ahlam A AbuElkhair, Ghislaine D Mbolo, Benjamin J Buckley, Nefyn H Williams, Deirdre A Lane

**Affiliations:** 1 Department of Cardiovascular and Metabolic Medicine, Institute of Life Course and Medical Sciences, University of Liverpool, Liverpool, UK; 2 Cardiac Rehabilitation and Research Centre, Physical Therapy Department, Chest Diseases Hospital, Kuwait Ministry of Health, Kuwait city, Kuwait; 3 Department of Physiotherapy, Faculty of Medical Technology, University of Tripoli, Tripoli, Libya; 4 School of Medicine, Institute of Life Course and Medical Sciences, University of Liverpool, Liverpool, UK; 5 Liverpool Centre for Cardiovascular Science, University of Liverpool, Liverpool John Moores University, and Liverpool Heart and Chest Hospital, Liverpool, UK; 6 Cardiovascular Health Sciences, Research Institute of Sport and Exercise Sciences, Liverpool John Moores University, Liverpool, UK; 7 Department of Primary Care and Mental Health, Institute of Population Health, University of Liverpool, Liverpool, UK

**Keywords:** Cardiovascular Disease, REHABILITATION MEDICINE, Coronary heart disease, Ischaemic heart disease, Exercise, Health Education

## Abstract

**Objective:**

To identify and synthesise the barriers and facilitators influencing the uptake, adherence and completion of cardiac rehabilitation (CR) programmes among underserved populations globally.

**Design:**

Mixed-methods systematic review.

**Data sources:**

MEDLINE (Ovid), PubMed, CINAHL, PsycINFO and Scopus were searched from 1 January 2000 to 9 September 2025. Reference lists and grey literature were also screened.

**Eligibility criteria for selecting studies:**

Qualitative, quantitative and mixed-methods studies examining barriers and/or facilitators to CR or physical activity participation among adults with cardiovascular disease from underserved populations (eg, women, ethnic minority groups and individuals with low socioeconomic status). Conference abstracts, editorials and studies without full text were excluded.

**Data extraction and synthesis:**

Study selection, data extraction and quality appraisal were conducted independently in duplicate. Methodological quality was assessed using the Mixed-Methods Appraisal Tool. Qualitative findings were synthesised thematically and mapped to the Capability, Opportunity, Motivation-Behaviour model and Theoretical Domains Framework. Quantitative findings were summarised using frequency-based vote counting.

**Results:**

85 studies (54 quantitative, 22 qualitative and 9 mixed-methods studies) involving 228 660 participants were included. Frequently reported barriers included comorbidities and fatigue, limited awareness of CR, communication difficulties, long travel distance, transportation and cost barriers, time constraints and inconsistent referral practices. Key facilitators included structured or automatic referral, health professional recommendation, family and social support, perceived physical and psychological benefits, improved self-efficacy and flexible delivery models such as home-based, telehealth or hybrid programmes.

**Conclusions:**

Barriers and facilitators to CR participation among underserved populations operate across individual, social and health system levels. Strategies that incorporate systematic referral, patient education, behavioural support and flexible delivery models may improve equitable access and sustained engagement with CR.

**PROSPERO registration number:**

CRD42024502384

**STRENGTHS AND LIMITATIONS OF THIS STUDY**

- This review integrates qualitative and quantitative evidence from 85 studies across diverse international settings.
- Findings were interpreted using the Capability, Opportunity, Motivation-Behaviour model and Theoretical Domains Framework to support theory-informed understanding of behavioural determinants.
- Considerable heterogeneity in study designs, populations and outcome measures precluded meta-analysis and limited standardised quantitative synthesis.
- Inconsistent reporting of ethnicity and socioeconomic indicators limited detailed subgroup comparisons.

## Introduction

 Cardiovascular disease (CVD) remains a leading cause of death globally, responsible for an estimated 19.8 million deaths in 2022, representing an 18.7% increase since 2010.^1 2^ Across Europe, CVD accounts for 45% of all deaths, with substantial disparities in mortality rates between Western and Eastern European countries.^3^ According to the British Heart Foundation, around 7.6 million people in the UK are living with heart disease and CVD.^4^ Despite advancements in treatment, individuals with CVD experience significant burden in terms of morbidity, hospitalisation and reduced quality of life.^5^

Cardiac rehabilitation (CR) programmes are well-established, evidence-based interventions designed to mitigate this burden through structured exercise, education and psychosocial support.^6 7^ CR has been shown to reduce mortality and hospital readmissions while improving physical and mental health outcomes.^8 9^ These programmes, often group-based, encompass a range of interventions such as exercise training, smoking cessation support, dietary counselling, medication education and psychological assistance.^10^

The efficacy of CR programmes in patients with coronary heart disease (CHD) has been extensively evaluated. A recent Cochrane review of participants with CHD (85 studies, 23 430 participants)^11^ highlighted the short-term benefits (6–12 months) of CR in reducing myocardial infarction (RR 0.72, 95% CI 0.55 to 0.93) and all-cause hospitalisation (RR 0.58, 95% CI 0.43 to 0.77). However, there was no statistically significant difference between groups for all-cause mortality (RR 0.87, 95% CI 0.73 to 1.04). Long-term benefits (>3 years), particularly in exercise-based CR, were evident in the reduction of cardiovascular mortality (RR 0.58, 95% CI 0.43 to 0.78) and myocardial infarction (RR 0.67, 95% CI 0.50 to 0.90), in addition to improvements in health-related quality of life.^11^

Despite the positive impact on patient outcomes, significant challenges persist in achieving equitable participation and completion rates among eligible patients,^11^ with participation rates ranging from 50% to 70%.^12^ Moreover, adherence to CR declines over time, with attrition ranging from 30% to 60%.^12^ Clear gender disparities are apparent, with women comprising<15% of participants in recent CR trials, despite bearing a substantial proportion of the global burden of CVD.^11 13^ Women often encounter caregiving responsibilities, low self-perceived need for CR and limited support from healthcare professionals.^14^ Ethnic minority groups also face barriers to CR participation, with evidence from the USA demonstrating substantially lower CR use among non-white patients compared with white patients (7.8% vs 19.6%).^13 15^ Further, these groups may experience language barriers, limited cultural tailoring of services and mistrust in the healthcare system.^16^ People from lower socioeconomic status (SES) backgrounds report practical obstacles such as transportation difficulties, work commitments and inadequate access to CR services, particularly in rural areas.^17^ Several reviews have examined the barriers and facilitators to CR among the general population of cardiac patients,^18–20^ yet these often overlook the specific challenges and enablers experienced by underserved groups. In addition, existing reviews have rarely integrated qualitative and quantitative evidence^21 22^ or applied behaviour change theory^23^ to understand determinants of CR participation among underserved populations.

To provide a theory-informed understanding of behavioural determinants influencing CR participation, this review was guided by the Capability, Opportunity, Motivation-Behaviour (COM-B)^24^ model and the Theoretical Domains Framework (TDF).^25–27^ Accordingly, this systematic review aimed to identify the barriers to, and facilitators of, joining, adhering to or engaging with CR programmes among patients from underserved groups.

## Methods

### Design

This mixed-methods systematic review integrated qualitative and quantitative evidence to address complex health and public health questions and to enhance the relevance of systematic reviews for informing practice.^28 29^ The Preferred Reporting Items for Systematic Reviews and Meta-Analyses (PRISMA) guidelines^30^ were followed in the reporting of this review, and the protocol was prospectively registered with PROSPERO (CRD42024502384).

### Search strategy

The following bibliographic databases were searched from 1 January 2000 to 9 September 2025: MEDLINE (Ovid), PubMed, CINAHL (EBSCO), PsycINFO and Scopus. The review aimed to include contemporary CR programmes; therefore, the search was restricted to studies published from the year 2000 onwards. Since 2000, CR programmes expanded to include alternative models and settings in addition to the conventional components (exercise training, educational sessions, dietary advice, smoking cessation and psychological support).^31^ The development of search terms and the implementation of the search strategy were supported by an information specialist. Combinations of Boolean operators, proximity operators, truncations and wildcards were used to search for medical subject headings (MeSH) terms or keywords and their synonyms such as “Cardiac Rehabilitation”, “Physical Activity”, “Exercise”, “cardiovascular disease”, “Barriers”, “Facilitators” and “Underserved/under-represented groups”. The full search strategy is available in online supplementary table 1. The search terms were adapted for use with the other bibliographic databases. In addition, a comprehensive search of grey literature, guidance, policy documentation and systematic reviews was performed, and their reference lists were examined for additional publications relevant to this review.

### Study selection

Eligibility criteria were defined according to population, study design, intervention and outcomes. Published literature, including qualitative, quantitative (randomised controlled trials (RCTs), non-RCTs, prospective cohort studies, cross-sectional studies and observational studies only), and mixed-method studies, was eligible. Conference abstracts, case studies or reports, commentaries and editorial articles were excluded. Studies without full-text publications were excluded. References were managed using EndNote X9 for Windows (Clarivate Analytics), while the screening process was supported by the online platform Rayyan (Qatar Computing Research Institute). Duplicate records were identified through a combination of electronic tools (EndNote and Rayyan) and manual searching. Title and abstract screening was conducted independently by two reviewers (HH and AAE), followed by full-text screening conducted independently by HH, AAE and GM. Any disagreements regarding study eligibility were resolved through discussion, with a third reviewer consulted when necessary (DL, NW and BB).

Studies including adults diagnosed with CVD, such as myocardial infarction, heart failure or those who had undergone coronary revascularisation procedures were eligible for inclusion (online supplementary table 2). All included studies described interventions as CR programmes consistent with the British Association for Cardiovascular Prevention and Rehabilitation standards, encompassing structured exercise training with one or more core components, such as health behaviour change and education, lifestyle risk factor management, psychosocial support, dietary advice or smoking cessation.^10^ Interventions involving physical activity alone or in combination with one or more additional component were eligible for inclusion.

The review prioritised underserved populations, including women, ethnic minority groups or individuals from lower SES backgrounds and other socially disadvantaged populations, due to their lower likelihood of engaging in or completing CR programmes.^32^ Eligible studies either focused exclusively on one or more underserved populations or included these groups within a broader CR sample, provided that their representation within the study population was reported or could be reasonably identified from the study context. Detailed definitions and criteria for the identification and inclusion of underserved populations are provided in online supplementary table 3. Where underserved status was not explicitly reported, classification was based on predefined indicators reported by study authors, including recruitment from socioeconomically deprived communities, low-income populations, predominantly ethnically diverse populations or other settings associated with social disadvantage.

Studies conducted across a variety of healthcare and community settings, including primary care, secondary care, community services, leisure centres, third-sector organisations, commercial providers, centre-based programmes, home-based programmes or a combination of both, were eligible for inclusion. Studies from low-income, middle-income and high-income countries were also considered.

### Quality assessment of included studies

The quality of the included studies was independently appraised by two authors (from HH, AAE and GM) using the Mixed-Methods Appraisal Tool (MMAT).^33^ The MMAT is specifically designed to facilitate the appraisal of heterogeneous study types within a single review framework, ensuring consistency and rigour in quality assessment. The MMAT (V.2018) comprises two initial screening questions followed by five methodological criteria, each rated as ‘Yes’, ‘No’ or ‘Can’t tell’. Notably, it does not generate a composite score; instead, it facilitates a detailed evaluation of each study’s methodological strengths and limitations. Study quality was not used as an exclusion criterion but was considered when interpreting and discussing the findings.

### Data extraction and data synthesis

A data extraction tool was developed using Microsoft Excel to systematically collect key characteristics from the included studies, comprising study authors, publication year, study design, country, methodology, data analysis methods, sample size, participant diagnosis, inclusion and exclusion criteria, as well as demographic variables such as sex, ethnicity, educational level and SES. Due to substantial heterogeneity in study designs, populations, settings and outcome measures, quantitative meta-analysis was not considered appropriate. Heterogeneity was assessed through examination of variability in study design, participant characteristics, underserved population definitions, intervention delivery models, outcome measures and methods used to assess barriers and facilitators. Given the substantial methodological and clinical heterogeneity across included studies, statistical pooling and meta-analysis were considered inappropriate.

Qualitative data were analysed and synthesised using an inductive approach following the framework proposed by Thomas and Harden’s method of thematic synthesis.^34^ The reported themes, sub-themes and supporting participant quotations were extracted and documented verbatim as presented by the original authors, then organised into broader themes that captured key barriers and facilitators across included studies. Emergent themes were subsequently categorised deductively within the framework of the COM-B^24^ model and the TDF.^25–27^ This framework was selected a priori because it provides a comprehensive and theoretically informed framework for understanding health-related behaviours. The COM-B model conceptualises behaviour change as contingent upon an individual’s capability, opportunity and motivation.^24^ The TDF, which complements and expands upon the COM-B model, comprises 14 domains designed to provide a more granular understanding of the mechanisms influencing evidence-based behavioural change^25–27^ (online supplementary figure 1). Together, these frameworks have been widely used to identify barriers and facilitators to healthcare participation and to inform the development of behaviour change interventions.

The emergent themes were subsequently mapped to the components of the COM-B model and the corresponding domains within the TDF. *Capability* corresponded with the TDF domains of *knowledge, skills, and memory, attention and decision processes*; *Opportunity* with *environmental context and resources and social influences;* and *Motivation* with *emotion, beliefs about capabilities, beliefs about consequences, social/professional role and identity, reinforcement, intentions and goals*.

To gauge the relative prominence of each theme, frequency analysis was employed to determine how often specific barriers and facilitators were reported across included studies.^23 35^ Frequency counts were used to describe the prevalence of reported barriers and facilitators across studies and were not interpreted as measures of their relative importance or influence on CR participation. Given the substantial number of included quantitative studies, a vote counting approach was employed to extract and summarise the reported barriers and facilitators within each study. This approach was guided by recommendations in the Cochrane Handbook.^36^ Two reviewers (AAE and GM) independently reviewed each included study and recorded identified barriers and facilitators in a predesigned Excel spreadsheet. A third reviewer (HH) verified and refined the extracted data, grouping similar items into broader themes and subthemes to support frequency analysis and ensure consistency.

### Patient and Public Involvement

Patients and/or the public were not involved in the design, conduct, reporting, or dissemination plans of this systematic review.

## Results

After removal of duplicate records, 28 793 study titles and abstracts were screened, resulting in the selection of 150 studies for full-text assessment. The study selection process is presented in the PRISMA flow diagram (online supplememtary figure 2). Following review of full texts, 85 studies were included based on the predefined criteria. 65 studies were excluded, and reasons for exclusion at the full-text stage are summarised in online supplememtary table 4.

### Study characteristics

#### Study types

Of the 85 studies included, 22 were qualitative,^37–58^ 9 were mixed-methods^9 59–66^ and 54 were quantitative studies^14 17 67–118^ (online supplementary table 5). All 22 qualitative studies used interviews (semi-structured in 20 studies^37–41 43–51 53–58^) to collect data. Of the nine mixed-methods studies, six used a convergent or explanatory sequential mixed-methods design, integrating retrospective cohort analyses or RCTs with thematic analysis from semi-structured interviews or focus groups.^9 60 62 63 65 66^ The remaining three studies^59 61 64^ combined structured interviews, patient surveys and qualitative interviews to explore barriers and facilitators to CR participation.

The 54 quantitative studies represented a diverse range of methodological designs. Cross-sectional designs were the most prevalent (n=21),^14 17 67 71 72 77 82–84 86 89 90 92 94 95 99 103 104 111 112 115^ followed by prospective cohort studies (n=10).^74 76 80 87 96 98 100 109 114 117^ Seven studies used RCT designs,^68 78 88 93 101 107 118^ six employed non-RCT or quasi-experimental designs^75 79 85 97 106 108^ and six were retrospective cohort studies.^69 73 91 102 110 116^ Two employed a prospective psychometric validation design,^70 113^ one used a case–control design^81^ and one was a cross-sectional multicohort study.^105^

#### Demographic characteristics

The included studies comprised 228 660 participants, of whom 99 695 (44%) were women. One study (n=9490)^91^ did not report the specific breakdown by sex, although sex was included as a covariate in analyses. These participants were included in the total sample size but excluded from the calculation of the overall proportion of female participants.

Of the 85 included studies, 44 (52%)^14 17 37 38 40–42 44–47 49–51 53 54 56 59 60 62–65 69 73 75 77 83 85–88 92 95 99–101 106 107 110 111 116–118^ explicitly reported participants’ ethnicity, while 14 (16%)^39 66–68 71 72 74 76 79–81 96 97 109^ included individuals from ethnic minority backgrounds, but did not report the exact distribution. The remaining 27 studies (32%)^9 43 48 52 55 57 58 61 70 78 82 84 89–91 93 94 98 102–105 108 112–115^ did not provide any information on participants’ ethnicity.

47 studies (55%)^9 14 17 37 42 43 48–53 55 57–62 67 70–72 76 77 82–84 87 89 90 92 95 98 99 102–106 108 111–115 117^ reported SES-related indicators, such as income, educational attainment or area-level deprivation. Another 15 studies (18%)^38 39 63 66 68 74 78–80 85 91 93 94 116 118^ were classified as including individuals from low-SES backgrounds, inferred through recruitment from publicly funded health services or low-income geographic areas. However, 23 studies (27%)^40 41 44–47 54 56 64 65 69 73 75 81 86 88 96 97 100 101 107 109 110^ did not provide any SES-related information.

#### Geographical distribution

Most studies were conducted in high-income countries, with the USA contributing the largest number (n=23),^56 59 64 65 69 72 73 75 80 82 85 92 94 95 99 101 103 105 110 111 116–118^ followed by Canada (n=14),^17 39 44 45 47 51 52 68 74 78 83 87 91 106^ the UK (n=8),^37 38 40 41 46 60 63 88^ and Australia (n=5),^9 43 49 62 104^ and one study was conducted in New Zealand.^86^ 15 studies were conducted in Asia (China n=6,^55 57 58 61 76 115^ Iran n=3,^71 97 108^ Japan n=3,^81 96 109^ Malaysia n=1,^77^ Singapore n=1,^100^ Indonesia n=1^53^) and four from the Middle East (n=1 each from Saudi Arabia,^54^ Israel,^79^ Jordan^67^ and Qatar^42^).

Within Europe, countries represented included Switzerland,^114^ Greece,^70^ Czech Republic,^113^ Germany and the Netherlands.^112^ Two studies were multinational studies,^14 107^ while Sweden,^48 90^ Denmark^66 84 93 98^ and Spain^50 89^ also contributed data.

### Quality assessment of the included studies

Overall, the studies demonstrated moderate to high methodological quality, with most (approximately 87%) meeting at least four of the five MMAT criteria. Only a small proportion (13%) exhibited notable methodological weaknesses.^64 68 75 78 117^

Among the 54 quantitative studies, the majority fulfilled criteria concerning clear research questions, appropriate measurement tools and alignment between the study objectives and analytical methods. However, some of the studies^17 61 68 72 75 78 83–86 88 93–97 99–101 103 106 108 112–117^ received ‘Can’t tell’ or ‘No’ ratings for one or more items, primarily relating to the management of potential confounders or the representativeness of recruited samples.

The 22 qualitative studies displayed strong coherence between research questions, methodological approaches and interpretations. All studies satisfied all five MMAT criteria, indicating robust methodological quality. Each study displayed strong alignment between research questions, data collection methods and interpretation, with findings well-substantiated by the evidence base. Collectively, these results indicate consistently high methodological quality. The nine mixed-methods studies demonstrated generally sound integration of quantitative and qualitative components, with most offering a clear rationale for methodological triangulation and appropriate synthesis of findings. However, two studies^9 64^ included isolated ‘Can’t tell’ responses concerning the depth of integration. One of these studies^64^ additionally noted minor inconsistencies between data sources. Overall, the included studies demonstrated moderate to high methodological quality across designs.

### Barriers and facilitators to CR uptake and adherence

Barriers and facilitators were organised according to the COM-B components and corresponding TDF domains, as described in the ‘Methods and illustrated in online supplementary figure 1.

#### Barriers to cardiac rehabilitation participation

31 studies contributed to the qualitative synthesis, comprising 22 qualitative studies and the qualitative components of 9 mixed-methods studies. Three studies did not identify any barriers.^39 53 55^

For the quantitative synthesis, data were extracted from 63 studies (54 quantitative, 9 mixed-methods studies). Out of these studies, 58 studies identified barriers.^9 14 17 60–62 64–88 90–100 102–109 111–118^ These findings were synthesised to produce a unified understanding of reported barriers and facilitators.

##### Barriers associated with Capability

###### Qualitative synthesis

Within the *physical capability* component (TDF: Skills), the most prevalent barrier reported was the *presence of comorbidities*, identified in 17 studies.^9 37 40 42 45 46 48 49 51 52 56 58–61 63 65^ Experiencing tiredness or weakness was also commonly cited (11 studies) as a limitation to CR participation.^37 42 45 49–52 58 60 61 63^

Within the *psychological capability* component (TDF: Knowledge), the most frequently reported barrier was a *lack of knowledge regarding CR or physical activity* within 11 studies.^9 45 47 49 51 54 58 59 62 65 66^ For the TDF domain of Memory, attention and decision processes, the most frequently reported issue was *communication difficulties* (seven studies)^9 40 41 43 54 61 66^ (figure 1).

**Figure 1:**
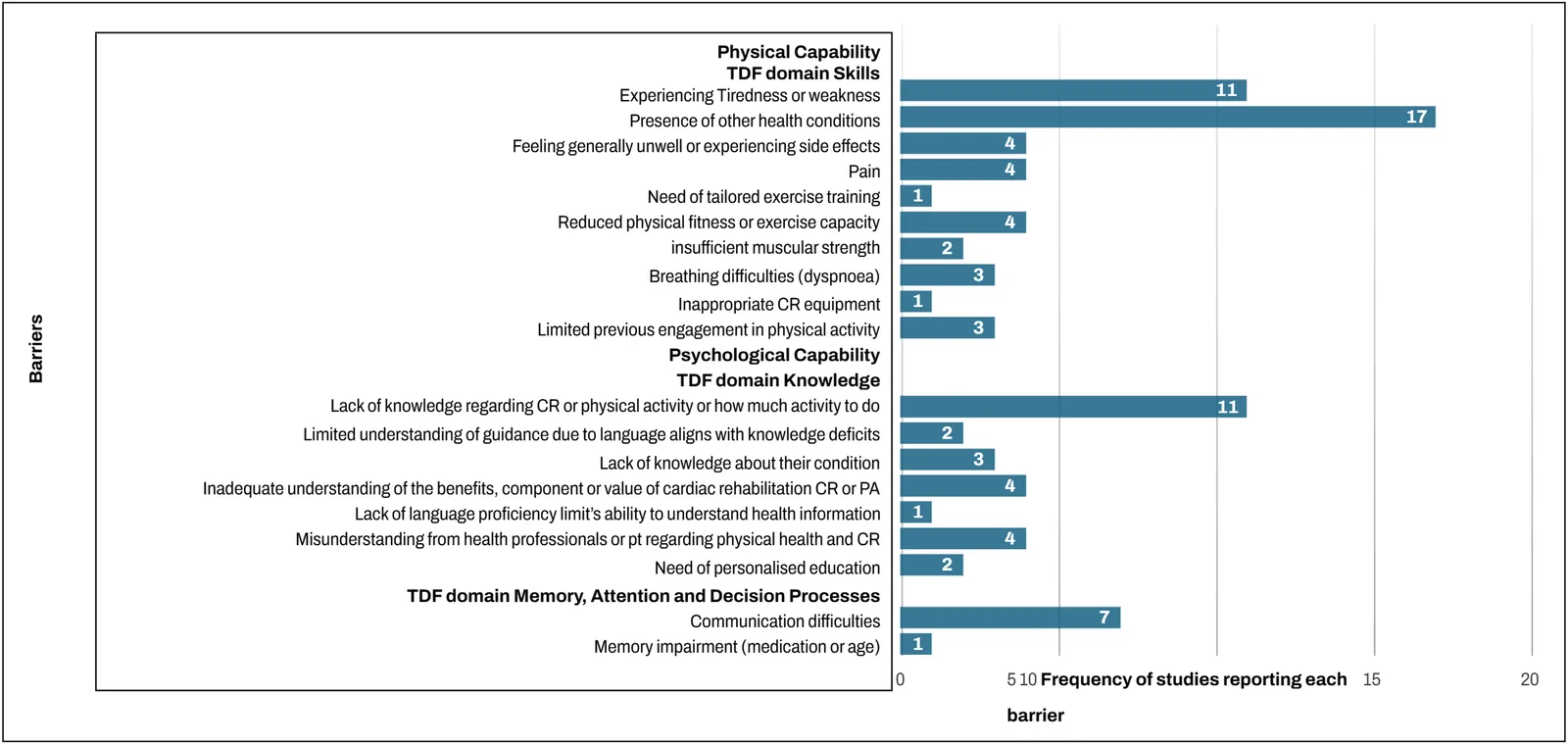
Barriers associated with *Capability* component identified from *qualitative data*. Bold headings represent Capability, Opportunity, Motivation-Behaviour (COM-B) components, while bold subheadings represent the corresponding Theoretical Domains Framework (TDF) domains. The relationship between COM-B components and TDF domains is illustrated in online supplemental figure 1. CR, cardiac rehabilitation; PA, physical activity.

###### Quantitative synthesis

Within the *physical capability* component (TDF: Skills), *other health problems (comorbidities*) were the most frequently reported barrier, identified in 16 studies,^14 66 77 82 83 94 98 105 108 109 113 114 118–121^ while * physical limitations* were reported in 12 studies.^67 74 75 90 92 97 99 103 112 114 117 120^

Within the *psychological capability* component (TDF: Knowledge), the *lack of awareness or understanding of CR* was the most frequent barrier, reported in 14 studies.^14 17 66 70 71 79 82 83 86 93 94 98 105 117^ The second most common barrier is *misconceptions about CR or perceiving it as unnecessary*, cited in six studies^66 67 83 84 107 117^ (figure 2).

**Figure 2:**
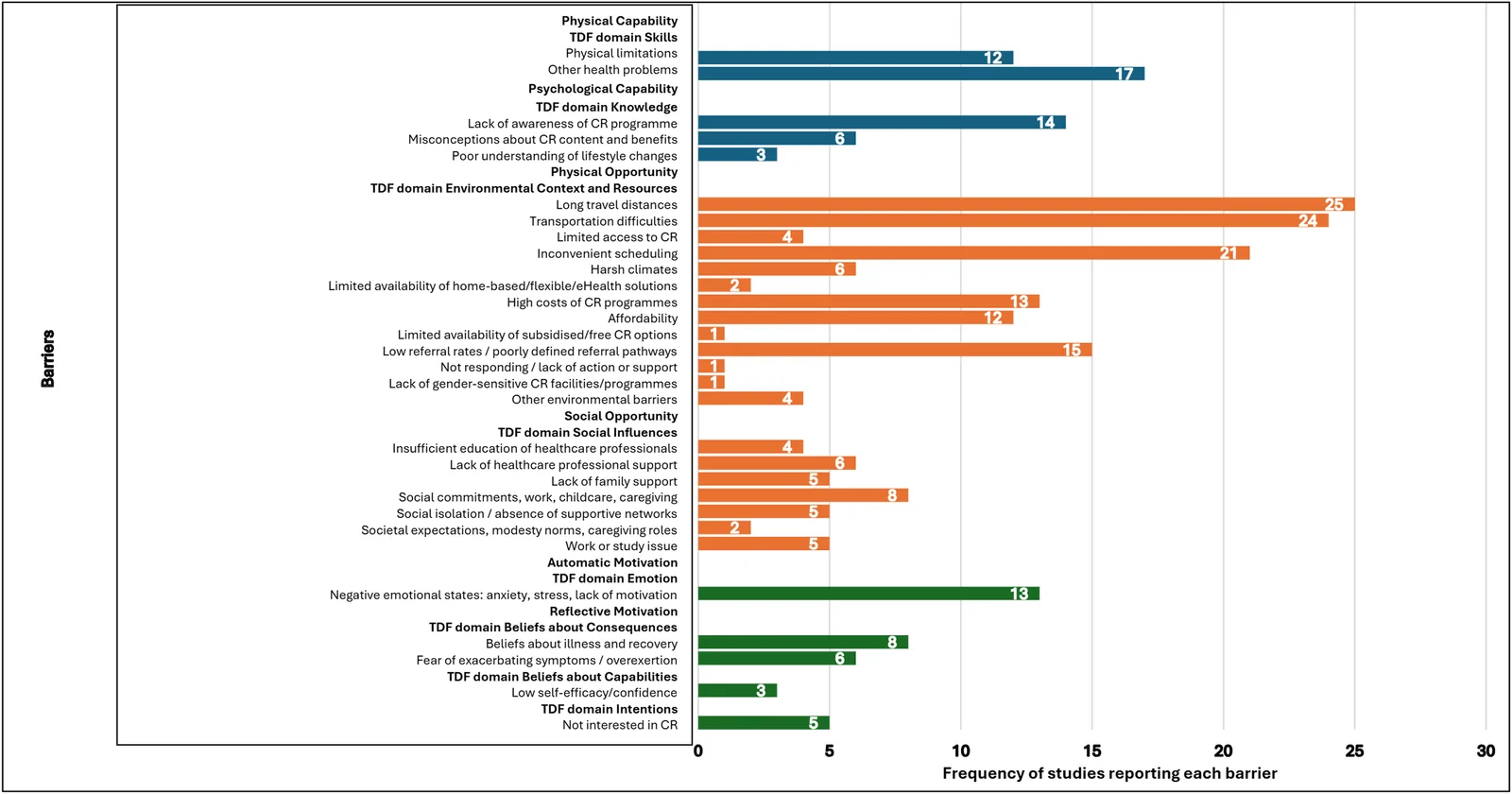
Barriers associated with *Capability, Opportunity and Motivation* component identified from *quantitative data*. Bold headings represent Capability, Opportunity, Motivation-Behaviour (COM-B) components, while bold subheadings represent the corresponding Theoretical Domains Framework (TDF) domains. The relationship between COM-B components and TDF domains is illustrated in online supplemental figure 1. CR, cardiac rehabilitation.

### Barriers associated with Opportunity

#### Qualitative synthesis

Within the COM-B component of *physical opportunity* (TDF: Environmental context and resources and social influences), the most frequently reported barriers were *limited availability of, or restricted access to, relevant facilities or structured programmes*, identified in 12 studies^9 37 44 46–49 51 54 59 63 64^ and *time constraints* in 10 studies.^9 40 44 46 47 49 51 52 58 63^ *Healthcare system barriers* were reported in nine studies,^9 41 43 46 47 54 60 61 63^ while *distance to the CR programme* was reported in eight studies.^9 40 46 47 49 59 63 65^

Within the *social opportunity* component (TDF: Social influences), *financial limitations, travel demands or occupational responsibilities* was the most frequently cited barrier to CR participation (14 studies),^9 42 44 46 49–52 57 58 61 63–65^ followed by *competing family duties, caregiving roles or social commitments* as another common barrier reported in 10 studies.^42 46 49 51 52 54 61 63–65^ Additionally, *healthcare professionals advised to reduce or stop physical activity* was reported as a barrier in seven studies^9 37 40 46 63–65^ (figure 3).

**Figure 3:**
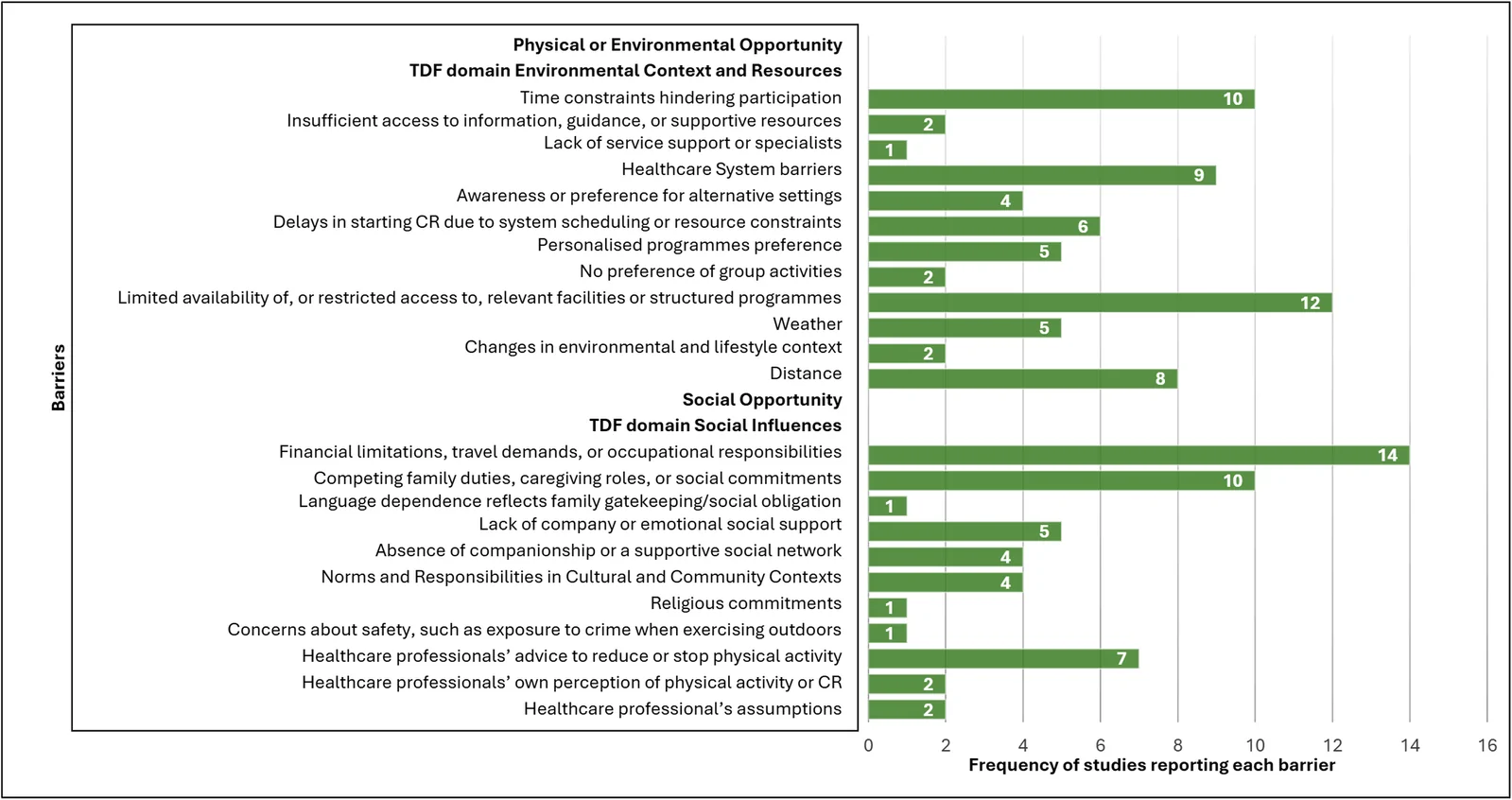
Barriers associated with *Opportunity* component identified from *qualitative data*. Bold headings represent Capability, Opportunity, Motivation-Behaviour (COM-B) components, while bold subheadings represent the corresponding Theoretical Domains Framework (TDF) domains. The relationship between COM-B components and TDF domains is illustrated in online supplemental figure 1. CR, cardiac rehabilitation.

#### Quantitative synthesis

Within the COM-B component of *physical opportunity* (TDF: Environmental context and resources and social influences), the most reported access barrier was *long travel distance*, identified in 25 studies.^14 17 70–72 74 75 77 80–83 85 87 92 95 96 98 99 102 105–108 113^ This was particularly challenging for individuals living in rural areas or without access to private or public transportation. The second most cited barrier was *transportation difficulties* (such as challenges with parking, associated costs or public transportation expenses), mentioned in 24 studies.^14 17 66–68 70–72 74 75 77 80 82–84 87 92 95 105–107 113 117 118^ The *lack of automatic or systematic referral* was also mentioned in 14 studies.^14 17 71 82 85–87 94 95 97 99 105 108 117^ This indicates significant variability in provider practices and highlights systemic gaps in CR referral pathways. Followed by other physical opportunity barriers like the *high cost of CR programmes* cited in 13 studies,^9 14 17 67 72 74 77 80 82 83 92 105 117^ and issues related to *affordability*, cited in also 13 studies.^14 17 32 71 75 91 93–95 103 116–118^

Within the *social opportunity* component (TDF: Social influences), the *social commitments, such as work, childcare or caregiving responsibilities*, was the most frequently cited barrier in this category, found in eight studies.^14 17 77 82–84 105 118^ This refers to competing family, cultural or community responsibilities that may limit an individual’s availability or prioritisation of. The second most frequent barrier was *lack of healthcare professional support*, identified in six studies,^14 77 82 83 105 106^ highlighting the role of insufficient encouragement or guidance from medical staff as a deterrent to programme participation (figure 2).

### Barriers associated with Motivation

#### Qualitative synthesis

In the motivation component of the COM-B model, several key barriers were identified across both *automatic and reflective motivation*, mapped to relevant TDF domains. For *automatic motivation* (TDF: Emotion), the most frequently reported barrier was *Low levels of intrinsic motivation*, cited in eight studies.^9 38 46 49 50 63 65 66^

Within *reflective motivation* encompassed a range of TDF domains. In the domain of Beliefs about consequences, the barrier *beliefs that physical activity is physically harmful* was reported most often, appearing in eight studies.^37 49 51 52 58 59 63 66^ The domain of Beliefs about capabilities was represented by *self-perceived limitations in interest, skill or suitability*, identified in 13 studies,^9 41 46 49 50 52 58–61 63 64 66^ followed by *low self-efficacy or confidence in one’s physical capabilities,* which was also frequently highlighted in relation to confidence, cited in eight studies.^37 42 45 51 52 58 65 66^ Under the goals and intentions domain, the barrier *belief that current activity levels are sufficient* was reported in eight studies^9 37 58 61 63–66^ (figure 4).

**Figure 4:**
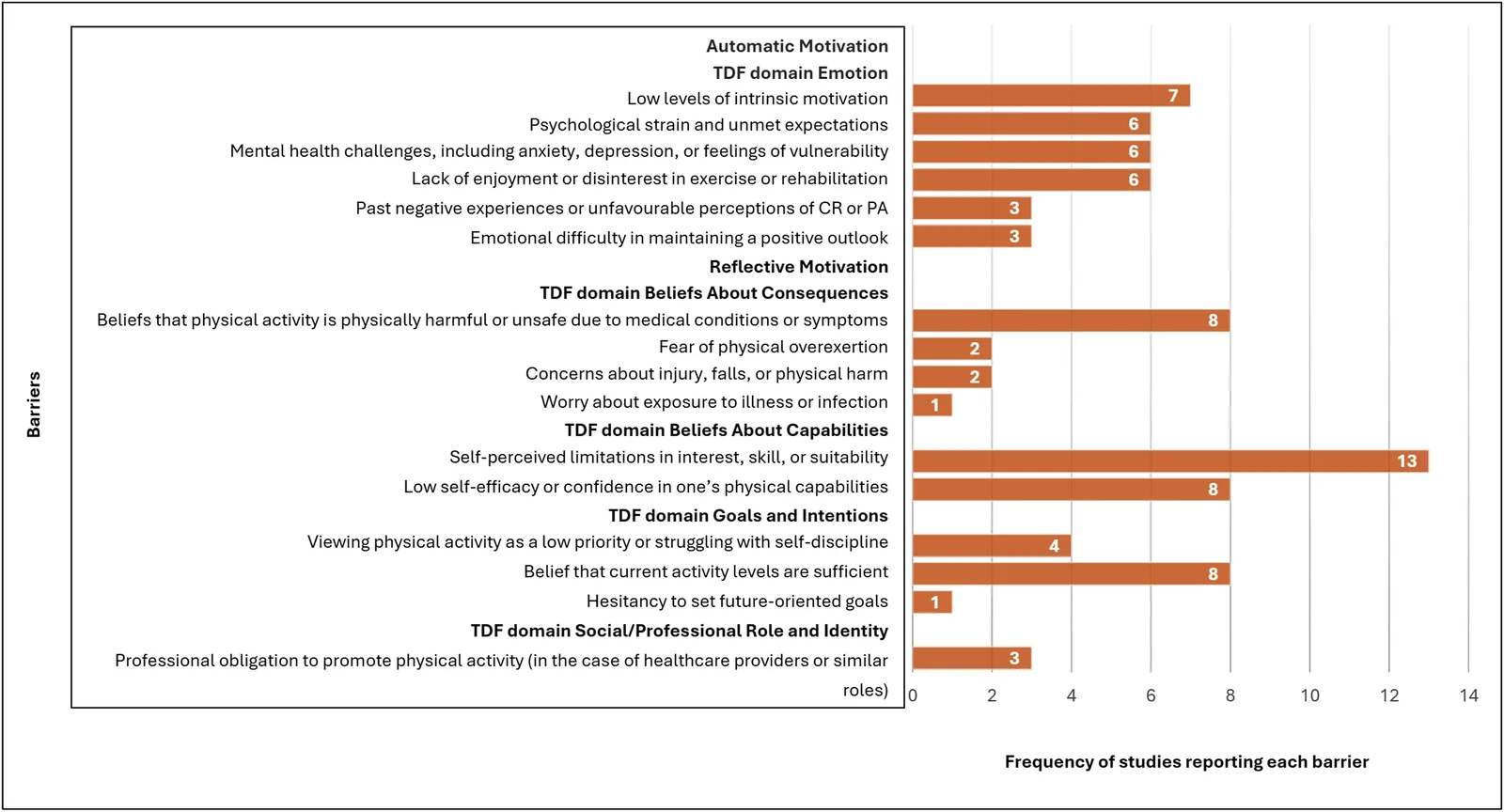
Barriers associated with *Motivation* component identified from *qualitative data*. Bold headings represent Capability, Opportunity, Motivation-Behaviour (COM-B) components, while bold subheadings represent the corresponding Theoretical Domains Framework (TDF) domains. The relationship between COM-B components and TDF domains is illustrated in online supplemental figure 1. CR, cardiac rehabilitation; PA, physical activity.

#### Quantitative synthesis

Within *automatic motivation* (TDF: Emotion), *negative emotional states including anxiety, depression or lack of motivation* was the most common barrier, appearing in 13 studies.^14 66 67 69 78 82 84 92 105 109 111 115 118^ These factors likely reduce psychological readiness to engage in CR, particularly during the early recovery phase.

Within *reflective motivation* (TDF: Beliefs about consequences), *beliefs about illness and recovery* was reported in eight studies.^14 66 76 78 82 83 105 106^ The domain of Beliefs about capabilities was represented by *low self-efficacy and confidence in one’s ability to complete CR*, which was reported in three studies.^17 67 104^ According to the Intentions domain, five studies^14 75 80 84 88^ reported that participants are *not interested in CR* (figure 2).

### Facilitators of cardiac rehabilitation participation

Of the 31 studies included from qualitative synthesis, 9 did not identify any facilitators that supported participants in initiating or completing CR programmes, or in maintaining regular physical activity.^9 43 44 50 52 58 61 63 65^ On the other hand, of the 63 quantitative studies, only 35 identified facilitators.^17 60 62–64 66–72 76–78 80 85 87 89 90 94–101 104 107 108 110 111 115 118^

#### Facilitators associated with capability

##### Qualitative synthesis

Within the *physical capability* component of the COM-B model (TDF: Skill), the most frequently reported facilitator was *absence of adverse effects*, supporting the maintenance of physical functioning (five studies).^37 55 56 59 60^

Regarding *psychological capability* (TDF: Knowledge), the most frequently reported facilitator was having *awareness of benefits or know how to promote physical activity* identified in 14 studies.^37 39 41 42 46 49 51 53 55–57 59 60 62^ In addition, *increased awareness of disease prevention* emerged in six studies as an enabler of participation.^42 46 51 53 55 59^

In the Behavioural Regulation domain of the TDF domain, the *development of structured routines or habitual behaviours* was the most frequently cited facilitator (six studies),^37 48 51 55–57^ suggesting that integrating CR or physical activity into regular daily practices may promote long-term adherence (figure 5).

**Figure 5:**
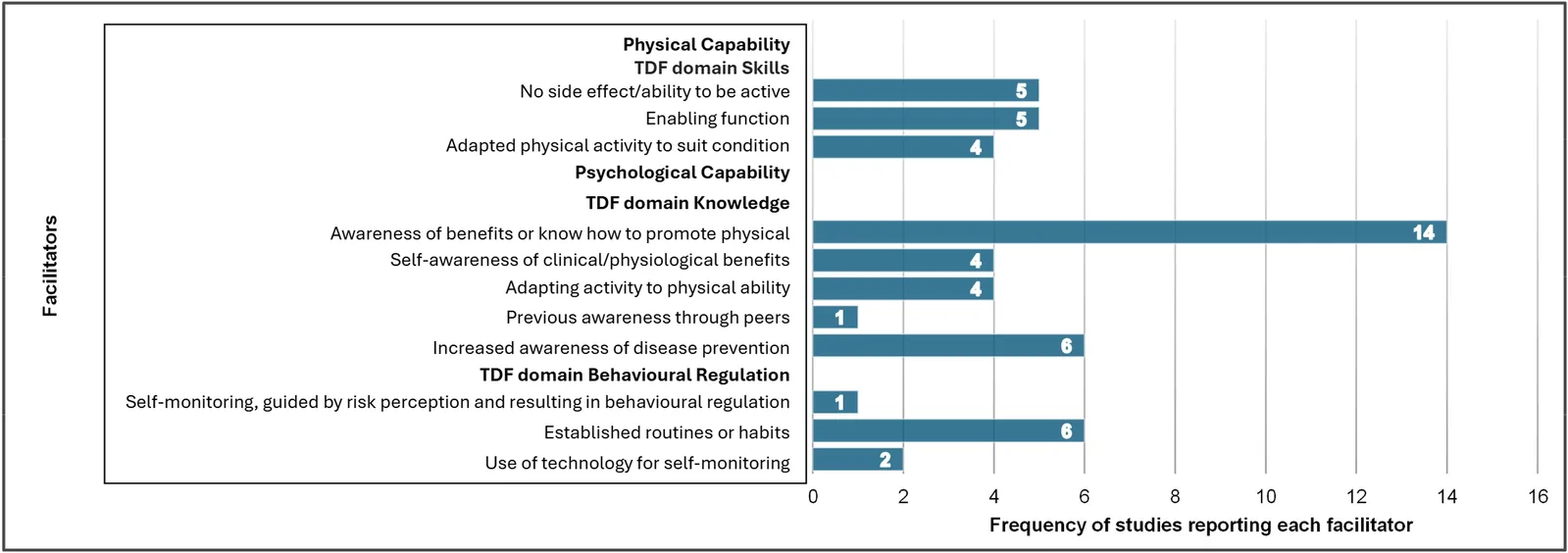
Facilitators associated with *Capability* component identified from *qualitative data*. Bold headings represent Capability, Opportunity, Motivation-Behaviour (COM-B) components, while bold subheadings represent the corresponding Theoretical Domains Framework (TDF) domains. The relationship between COM-B components and TDF domains is illustrated in online supplemental figure 1.

##### Quantitative synthesis

No quantitative studies identified facilitators corresponding to the *Physical Capability* component. However, *psychological capability* (TDF: Knowledge), *Education* was reported in four studies as a facilitator. In TDF: Behavioural Regulation, *monitoring of progress* was reported in three studies^60 66 80^ (figure 6).

**Figure 6:**
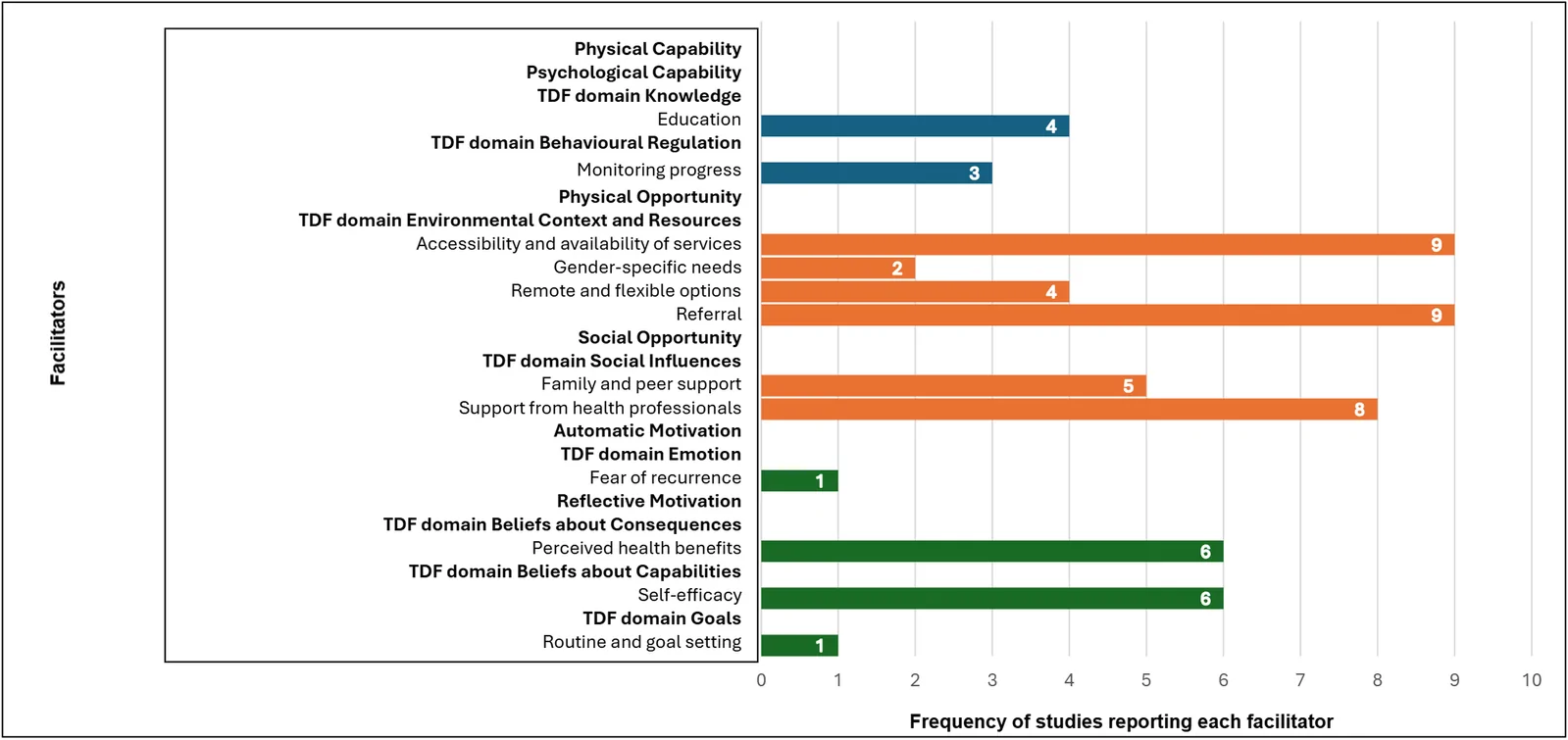
Facilitators associated with *Capability, Opportunity and Motivation* components identified from *quantitative data*. Bold headings represent Capability, Opportunity, Motivation-Behaviour (COM-B) components, while bold subheadings represent the corresponding Theoretical Domains Framework (TDF) domains. The relationship between COM-B components and TDF domains is illustrated in online supplemental figure 1.

### Facilitators associated with Opportunity

#### Qualitative synthesis

Within the *physical opportunity* component (TDF: Environmental context and resources and social influences), several key facilitators were frequently identified, including *environmental motivators* (11 studies)^37 45 46 51 53–57 59 62^ and the *ability to attend/access facilities or information* (7 studies).^39 41 42 51 54 60 62^

Within the *social opportunity* component (TDF: Social influences), the most frequently mentioned facilitator was *health professional advice, support and encouragement* identified in 18 studies.^37 39–42 45 46 48 49 51 53 56 57 59 60 62 64 66^ This was followed by *family or friend social support,* which was reported as a facilitator in 13 studies^37–40 42 48 49 53 54 56 57 62 64^ (figure 7).

**Figure 7:**
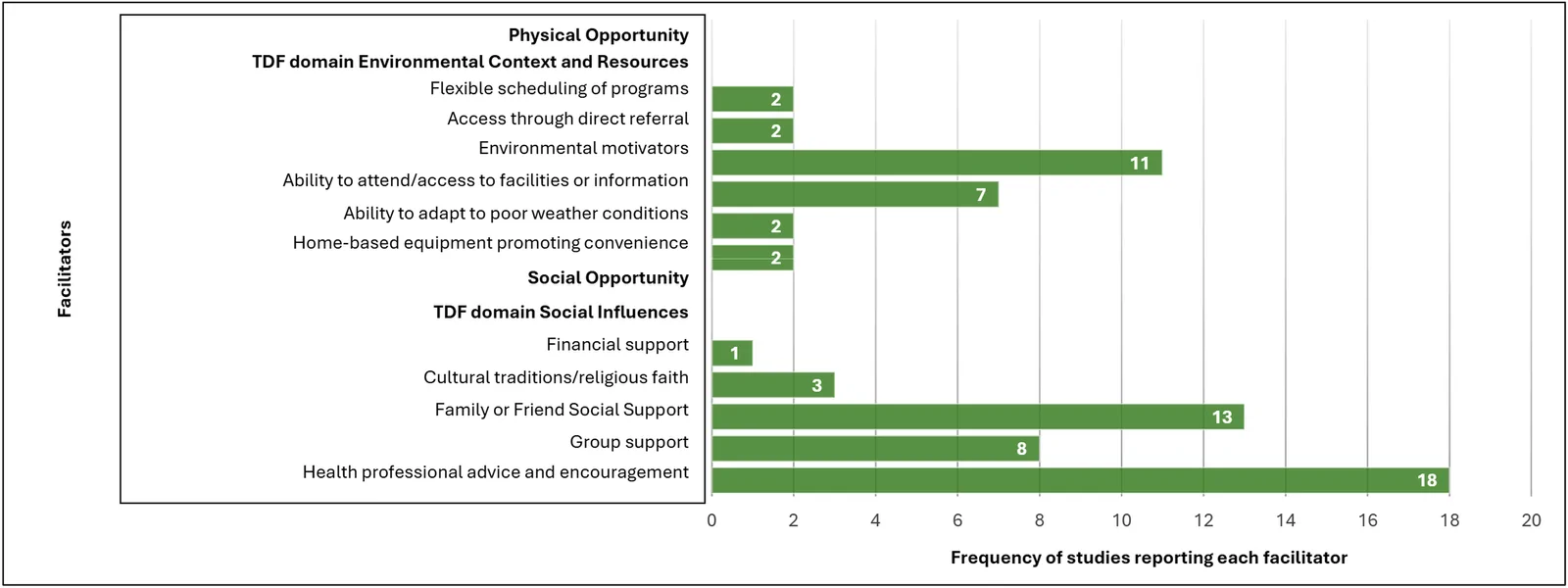
Facilitators associated with *Opportunity* component identified from *qualitative data*. Bold headings represent Capability, Opportunity, Motivation-Behaviour (COM-B) components, while bold subheadings represent the corresponding Theoretical Domains Framework (TDF) domains. The relationship between COM-B components and TDF domains is illustrated in online supplemental figure 1.

#### Quantitative synthesis

Within the *physical opportunity* component (TDF: Environmental context and resources and social influences), the two most frequently identified facilitators were *referral and accessibility and availability of services. Referral,* cited in nine studies,^60 63 72 80 97 99 102 108 110^ was reflecting the critical role of direct and systematic referral processes in encouraging participation. *Accessibility and availability of services* was also cited in nine studies,^66 68 70 71 94 96 104 111 118^ which included factors that help reduce logistical barriers and promote continued involvement. Other facilitators, though less frequently reported, included access to *remote and flexible options* (four studies).^62 104 107 118^

Within the *social opportunity* component (TDF: Social influences), *support from health professionals* was reported in eight studies^64 66 68 76 80 95 111 118^ and family or peer support in five studies,^64 72 77 78 115^ highlighting the importance of both professional encouragement and informal social networks in maintaining motivation and adherence (figure 6).

### Facilitators associated with Motivation

#### Qualitative synthesis

Within the motivation component of the COM-B model, a range of facilitators were identified across both automatic and reflective motivational processes, each aligned with relevant domains of TDF domains. In relation to *automatic motivation* (TDF: Emotion), the most frequently reported facilitator was *release stress and improve mental health* identified in 12 studies,^37 39 41 48 49 51 53 55–57 59 66^ followed by *links directly to affective response to activity*, noted in 11 studies.^37 48–51 53 55–57 59 62^ Under the Reinforcement domain of TDF, the most cited facilitator was *belief in the value of the intervention, reflected in perceived enhancements in quality of life, symptom relief and physical capability*, which appeared in 13 studies.^39 46 48 49 51 53 55–57 59 62 64 66^

Within *reflective motivation***,** several TDF domains were represented. In the domain of Beliefs about consequences, two facilitators, *experienced health improvements* reported in 11 studies,^39 48 49 51 53 55–57 59 62 64^ followed by *having physical or/and emotional benefits*, reported in 10 studies.^42 48 49 51 53 55–57 59 62^ Finally, for the domain of Beliefs about capabilities, the facilitators of *long-term autonomy as motivator*^37 39 41 42 48 49 53 55–57 59^ and *perceptions of those able or interested in participating*^37 39 41 47 51 53 55 57 59 60 64^ were each identified in 11 studies. Within the domain of Goals and intentions, *social planning* was recognised as a key facilitator in nine studies.^37 38 47 49 51 53 55 57 64^ Within the domain Social/professional role and identity*, religion and spiritual alignment with health behaviours* was reported in two studies.^42 54^ Participants described that culturally aligned care and faith-supported beliefs reinforced positive attitudes towards health, increased motivation to adopt healthier behaviours and encouraged engagement with CR programmes (figure 8).

**Figure 8:**
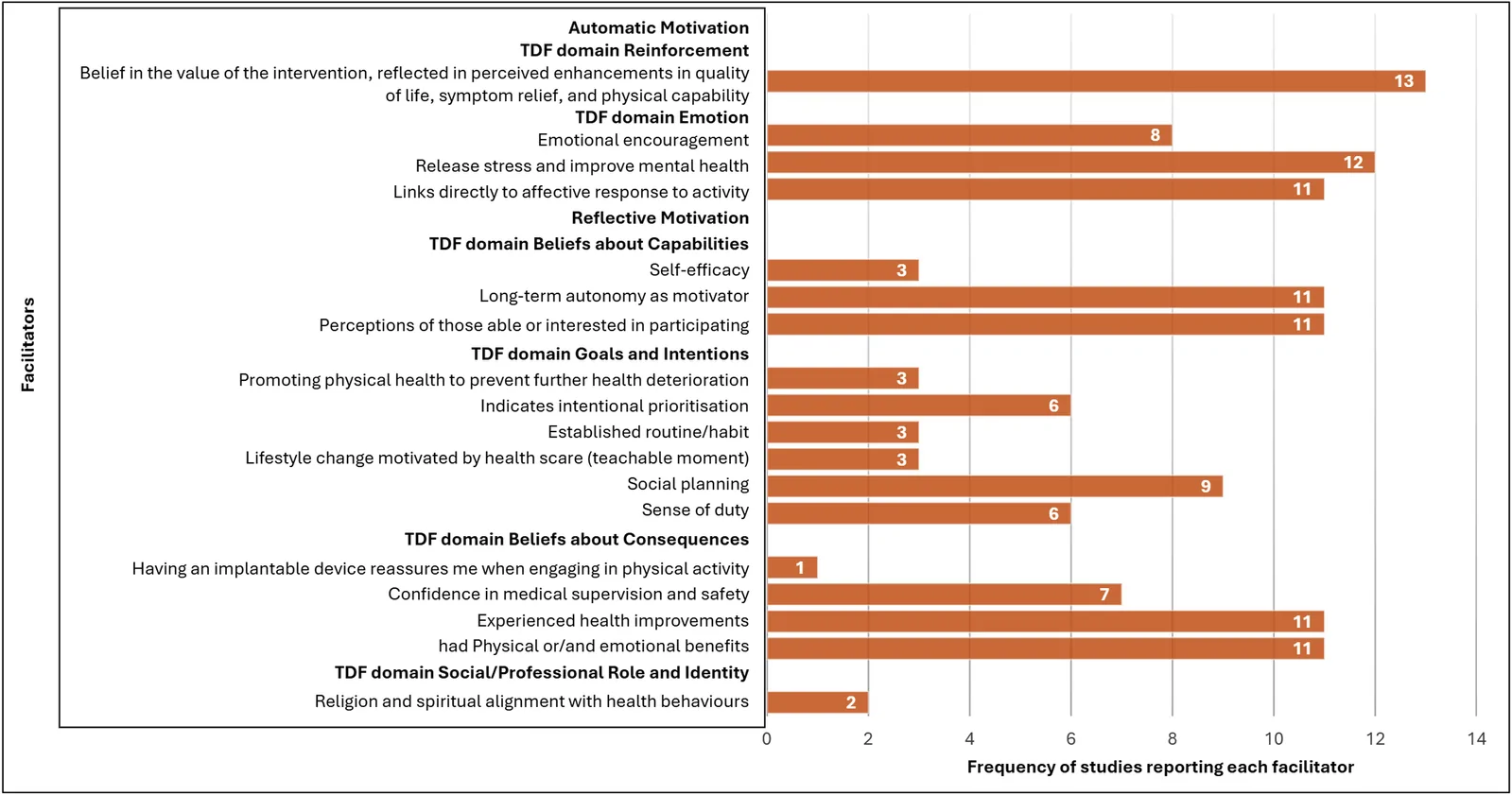
Facilitators associated with *Motivation* component identified from *qualitative data*. Bold headings represent Capability, Opportunity, Motivation-Behaviour (COM-B) components, while bold subheadings represent the corresponding Theoretical Domains Framework (TDF) domains. The relationship between COM-B components and TDF domains is illustrated in online supplemental figure 1.

#### Quantitative synthesis

Within *automatic motivation* (TDF: Emotion), *fear of recurrence* was reported in one study only.^64^ Within *reflective motivation* (TDF: Beliefs about consequences), *perceived health benefits* were cited in six studies^64 66 67 80 90 99^ as a motivating factor, as patients who recognised improvements in quality of life, symptom management or physical function were more likely to continue with CR programmes. The domain of Beliefs about capabilities was represented by *beliefs about one’s own capabilities,* such as confidence to engage in CR, appeared in six studies,^69 72 89 90 98 115^ suggesting that patients’ self-efficacy and perception of their own physical ability play a significant role in enabling ongoing participation. According to Intentions domain, *routine and goal setting* was reported in one study.^60^ (figure 6)

## Discussion

### Overview of main findings

This mixed-methods systematic review synthesised evidence from 85 studies to identify the key barriers and facilitators influencing participation and adherence to CR among underserved groups. Across both qualitative and quantitative evidence, consistent barriers and facilitators emerged across the COM-B components and corresponding TDF domains. Health-related limitations, including multimorbidity, low exercise tolerance and persistent fatigue, commonly constrained patients’ capability to attend or engage with CR. Limited awareness of CR and communication challenges (eg, unclear information at discharge) hindered enrolment, while logistical obstacles such as travel distance, transport difficulties, time constraints and costs reflected structural inequities in access. Referral system limitations, particularly inconsistent or passive referral practices, further impeded uptake. Psychosocial factors, including anxiety, low motivation and competing caregiving or employment responsibilities, also influenced non-participation, illustrating how capability, opportunity and motivation interact to influence CR participation and adherence.

Although many barriers were shared across underserved populations, some were more prominent within specific groups. Women more frequently reported caregiving responsibilities, competing family commitments and gender-related barriers to participation. Ethnic minority groups more commonly identified language barriers, communication difficulties and cultural influences affecting engagement, whereas individuals from lower socioeconomic backgrounds were more likely to experience financial constraints, transportation difficulties, limited access to services and digital exclusion. These findings suggest that although many determinants of CR participation are common across underserved populations, tailored strategies are likely to be required to address the distinct needs of different groups.

Conversely, facilitators demonstrated similar convergence across evidence types. A strong understanding of CR’s relevance and expected benefits, together with early perceived improvements in physical or emotional well-being, reinforced motivation to continue. Support from healthcare professionals, family and peers enhanced confidence and accountability, while flexible programme formats, especially home-based and hybrid models, addressed practical access barriers. Self-efficacy, enjoyment of physical activity and forming routine-based strategies were consistently linked with sustained participation. A small number of qualitative studies also highlighted the positive influence of culturally aligned or faith-supported health beliefs, while quantitative studies noted that structured referral systems and progress tracking could reinforce engagement.

### Interpretation in context

#### Capability: health status, skills and psychological readiness

Health-related limitations (comorbidities, fatigue) observed in this review mirror findings from contemporary cohorts in heart failure and those recovering from cardiac interventions, such as percutaneous coronary intervention or coronary artery bypass grafting, where baseline exercise capacity, disease severity, depression and symptom vigilance strongly shape participation trajectories.^122^ Both qualitative and quantitative findings consistently demonstrated that physical capability was primarily limited by multimorbidity, fatigue and reduced functional capacity, while psychological capability was constrained by poor knowledge of CR, misconceptions regarding exercise and communication difficulties. These findings are consistent with those of Bäck *et al*,^20^ who demonstrated that supportive interactions with physiotherapists during the acute phase helped patients develop confidence, reduce fear of exercise and improve readiness to participate in exercise-based CR, highlighting the importance of early professional reassurance and education in enhancing psychological capability. Furthermore, long-term telemonitoring trials have shown that adherence to prescribed activity declines over time and is predicted by age, heart failure severity, atrial fibrillation, baseline exercise behaviour and body mass index, underscoring the need to embed relapse-prevention supports to sustain long-term participation and physical activity.^123^ Psychological capability also emerged as an important determinant of participation, as fear of physical activity (kinesiophobia) is independently associated with avoidance and non-participation. These findings emphasise the importance of incorporating targeted psychoeducation and anxiety management into CR programmes.^124^

#### Opportunity: structural access and social context

The most common opportunity-related barriers identified across both qualitative and quantitative evidence, including travel distance, transportation difficulties, cost, time constraints and inconsistent referral pathways, are consistent broader rehabilitation literature demonstrating that rehabilitation services often remain service-centric, placing responsibility on patients to overcome access barriers.^125^ Patients report that options rarely fit employment, caregiving or mobility constraints, and that navigating access is framed as the individual’s responsibility.^125^ This individualised burden reflects wider structural and geographical inequities in rehabilitation provision, particularly in low-resource or rural settings.^126 127^ Both qualitative and quantitative findings highlighted inconsistent referral practices as an important opportunity-related barrier, suggesting that improvements to systematic referral pathways may increase equitable access to CR. Consistent with previous systematic reviews, flexible, home-based and hybrid models of CR have been shown to improve accessibility and support continued participation, particularly among individuals facing transportation or geographical barriers.^18 19 128^ However, the effectiveness of these approaches depends upon equitable access to technology and adequate digital literacy. These barriers are further intensified by socioeconomic deprivation, which introduces digital exclusion, financial strain, stigma and reduced social support. Such intersecting disadvantages constrain self-management capacity across long-term conditions and disproportionately affect individuals with multimorbidity, a profile common among CR-eligible patients.^125 129 130^

#### Motivation: beliefs, affect and behaviour change

Across both qualitative and quantitative evidence, motivational barriers were characterised by anxiety, fear of exercise, low self-efficacy and negative beliefs regarding illness and recovery, whereas facilitators included confidence in one’s ability to participate, perceived health benefits and positive reinforcement from CR participation.

Digital and behaviourally informed interventions may help address these motivational barriers by reinforcing goal setting, self-monitoring (defined as the systematic observation and recording of one’s own behaviours or progress towards behavioural goals) and feedback while simultaneously overcoming practical barriers such as travel distance, work commitments and caregiving responsibilities. Collectively, these findings indicate that although digital modalities can extend the reach of CR, their effectiveness depends upon equitable access to technology, adequate digital literacy, behaviourally informed intervention design and sufficient duration to consolidate long-term behaviour change. Addressing these barriers is essential to ensure that digital CR models do not inadvertently widen existing inequalities in access and participation.

Emerging evidence suggests that digital and behaviourally informed interventions can maintain physical activity following completion of centre-based CR. For example, the RehabPlus+ quasi-experimental trial demonstrated that personalised behavioural messaging sustained physical activity levels over 24 weeks, achieving outcomes comparable to supervised outpatient maintenance programmes.^131^

Despite these encouraging findings, a recent meta-analysis of internet-based CR RCTs reported no pooled superiority over usual care for psychological outcomes such as anxiety, depression or quality of life, underscoring the heterogeneity of digital interventions and the need for longer, theory-driven designs tailored to patient needs.^132^ Complementary approaches that address emotional and behavioural barriers, including mind–body tele-group formats such as tele–Qi Gong, have demonstrated feasibility, high attendance and patient-reported improvements in stress and sleep quality.^133^ Further, integration of an explicit therapeutic engagement model within standard CR has been shown to enhance medication adherence and patients’ sense of coherence compared with traditional educational approaches, highlighting the value of structured behavioural frameworks in promoting sustained self-management.^134^

Collectively, these findings indicate that while digital modalities can extend the reach of CR, their effectiveness depends on grounding design, incorporating psychological support and sufficient duration to consolidate long-term behaviour change.

### Comparison with previous systematic reviews

Many of the barriers and facilitators identified in this review are consistent with previous systematic reviews of CR participation in the general cardiac population.^18 19^ Common barriers, including travel distance, transportation difficulties, physical limitations, competing commitments and low referral rates, have been repeatedly identified as major determinants of CR uptake and adherence. Likewise, healthcare professional support, systematic referral pathways, family support and flexible or home-based delivery models have consistently been recognised as important facilitators of participation.^18 19^

However, the present review extends the existing literature by specifically focusing on underserved populations and synthesising mixed-method evidence using the COM-B model and the TDF. Although many determinants were shared with the general CR population, underserved groups experienced additional barriers that have received comparatively less attention, including language barriers, cultural and religious influences, caregiving responsibilities, financial hardship, limited access to culturally appropriate services and gender-specific needs. Importantly, these barriers often reflected intersecting forms of disadvantage, whereby individuals with multiple underserved characteristics experienced overlapping social, cultural and structural challenges to CR participation. These findings suggest that improving CR participation among underserved populations requires not only addressing traditional logistical barriers but also tackling broader social, cultural and structural determinants of access.

### Implications for practice

This review emphasises the importance of transitioning from standardised, centre-based CR models toward more inclusive, flexible and person-centred approaches, particularly for underserved groups. The findings support the potential role of flexible and digitally enabled CR models in addressing access barriers, particularly for individuals with multimorbidity, geographical constraints or competing life demands. Digital and remote programmes should be considered alongside centre-based services as one component of flexible, personalised CR delivery rather than a universal solution.

To enhance participation, CR services should adopt personalised, culturally responsive interventions that address patients’ beliefs, emotional readiness and social contexts. Systematic and automatic referral pathways must be strengthened to ensure equitable access. Additionally, integrating psychosocial support and behavioural strategies, both in-person and digitally, can enhance long-term engagement. Addressing these barriers through patient support, alternative delivery options and culturally appropriate materials may help reduce inequities.

Finally, future CR research should prioritise the consistent reporting of ethnicity and socioeconomic indicators to better understand and address disparities in access and outcomes. National health policies should view CR as a tool for reducing health inequalities and support its broader, more accessible implementation.

### Strengths and limitations

This review presents a comprehensive synthesis of barriers and facilitators to CR participation and adherence among underserved populations, drawing upon both qualitative and quantitative evidence across diverse settings. A notable methodological strength is the rigorous screening and data extraction process, which was conducted independently and in duplicate to minimise bias and ensure accuracy. By synthesising findings across 85 studies and incorporating diverse methodological designs, including cross-sectional surveys, RCTs, cohort studies and in-depth qualitative interviews, it offers a nuanced understanding of the multifaceted factors influencing CR participation and adherence. The use of the COM-B model and the TDF to structure and interpret findings enhances the theoretical rigour and allows for clearer translation into behaviour change interventions. Importantly, the review incorporated grey literature and studies published in non-English languages, thereby minimising publication and language bias and increasing the comprehensiveness and inclusivity of the evidence base. Furthermore, the inclusion of studies from a wide range of countries supports the broader applicability of the findings, while the specific emphasis on underserved groups addresses a recognised gap in the CR literature and provides valuable insights for equity-oriented policy and practice.

By integrating evidence across diverse methodologies and focusing specifically on underserved populations, the review extends existing literature that has typically examined these factors separately. Mapping findings onto the COM-B framework further enabled a theoretically informed understanding of how personal, social and structural determinants interact to shape CR participation.

Despite these strengths, several limitations should be acknowledged. First, while the review aimed to capture underserved populations, inconsistencies in how studies reported ethnicity, SES and other demographic indicators limited the ability to draw clear comparisons across subgroups. In some cases, the classification of participants as ‘underserved’ had to be inferred based on study context, rather than being explicitly stated. Although the search strategy incorporated broad terms relating to ethnicity, minority populations and underserved groups, it did not explicitly include the term ‘Indigenous’ or country-specific descriptors such as Aboriginal, First Nations, Native American or Māori. This reflected the international scope of the review, as terminology used to describe Indigenous populations varies across countries and healthcare contexts. Relevant studies including Indigenous populations, such as Aboriginal and Torres Strait Islander peoples in Australia and Māori populations in New Zealand, were identified and included. Second, the heterogeneity of study designs, settings and outcome measures posed challenges for standardised synthesis and precluded meta-analysis. Additionally, although quality appraisal was conducted, the methodological quality of included studies varied, particularly among qualitative studies where reflexivity and transparency were sometimes limited. Furthermore, the frequency with which barriers and facilitators were reported across studies does not necessarily reflect their relative importance or magnitude of effect on CR participation. The frequency analyses used in this review describe reporting patterns within the literature and should not be interpreted as measures of causal influence or effect size. Consequently, highly reported factors, such as travel distance or transportation barriers, may not necessarily represent the strongest determinants of participation.

The overall methodological quality of the included studies was moderate to high, yet some limitations were evident. Among quantitative studies, weaknesses commonly related to unmeasured or uncontrolled confounding, small sample sizes and limited representativeness of recruited cohorts, particularly in single-centre observational designs. Several studies lacked clarity on how missing data or attrition were handled, potentially introducing bias. Qualitative studies generally demonstrated strong coherence between research questions, methods and interpretation, though reflexivity and transparency in analytic processes were inconsistently reported. A minority of mixed-methods studies showed limited integration between components, which may have reduced the depth of synthesis across data types. These methodological constraints, while modest, should be considered when interpreting the weight of evidence and generalising findings.

Collectively, despite some heterogeneity and reporting limitations, the predominance of studies meeting four or more MMAT criteria supports confidence in the reliability and validity of the overall synthesis.

## Conclusions

This mixed-methods systematic review indicates that participation in CR among underserved populations is influenced by interacting capability, opportunity and motivation related determinants that operate across patient, healthcare system and societal levels. Findings highlight the need for CR services to adopt more flexible, person-centred and equitable approaches. Embedding behavioural frameworks, strengthening systematic referral pathways, and expanding hybrid or digital models may help to reduce inequalities and improve long-term engagement among underserved groups. Further research should evaluate the effectiveness of these approaches in real-world settings.

## Supplementary material

10.1136/bmjopen-2026-119214 online supplemental file 1

## Data Availability

Data may be obtained from a third party and are not publicly available. All data relevant to the study are included in the article or uploaded as supplementary information.
