# Supplementary material for: Barriers and facilitators of cardiac rehabilitation focusing on underserved groups: a mixed-methods systematic review
Source: BMJ Open. 2026 Oct 29;16(10):e119214. doi: 10.1136/bmjopen-2026-119214 (PMC13723823; doi:10.1136/bmjopen-2026-119214)
Supplement: Supplementary file 1 [file bmjopen-2026-119214.reviewer_comments.pdf]

PEER REVIEW HISTORY

BMJ Open publishes all reviews undertaken for accepted manuscripts. Reviewers are asked to complete a checklist review form and are provided with free text boxes to elaborate on their assessment. These free text comments are reproduced below.

ARTICLE DETAILS

Title (Provisional)

Barriers and facilitators of cardiac rehabilitation focusing on underserved groups: A mixed-methods systematic review

Authors

Humoud, Hanan; AbuElkhair, Ahlam A; Mbolo, Ghislaine D; Buckley, Benjamin; Williams, Nefyn; Lane, Deirdre A

VERSION 1 - REVIEW

**Reviewer**	**1**
**Name**	**Lee, Yonggu**
**Affiliation**	**Hanyang University Guri Hospital, Department of Internal Medicine**
**Date**	**27-Mar-2026**
**COI**	**None**

General comments

This mixed-method systematic review of 85 studies is to summarize the barriers and facilitators influencing cardiac rehabilitation participation among underserved population. Using COM-B model and TDF, the authors showed that barriers operate across multiple levels including patient-related factors (physical health, skills and psychological readiness), structural constraints (travel time, cost, limited access) and psychological determinants (low motivation) and factors such as systemic referral, healthcare professional support, social support, and flexible delivery models (telehealth CR) could be facilitators for CR.

This is a well-conducted, methodologically sound and well written review. By including 85 studies of various types, they minimized publication bias and covered a wider range of ethnic groups and languages. Also, they employed COM-B TDF to integrate those studies with different methodologies. However, the fact that they included so many different methodologies prevented them from performing quantitative analyses with some effect sizes or estimating at least the proportions of patients with the barriers or facilitators. Because the measurements frequently appeared in published studies often represent how easy to measure the variable rather than how important they are as determinants of a

phenomenon, we should bear in mind that a large number of studies regarding certain barriers/facilitators does not always mean their importance as factors in the clinical practices. For example, the long travel distance is the most frequently appeared barrier in the quantitative studies, but without the effect size of 1km travel distance increase to the CR initiation, the importance of the travel distance cannot be accurately estimated. Therefore, the authors should acknowledge the possibility that the plots might give some incorrect interpretation and impression to readers that the most important barrier is the travel distance and should try to clearly state this concern in the manuscript.

Specific comments

I only have minor comments without concerns.

Regarding Figure 6, "Side efect" is a spelling error. In the same figure, what does the "Specific… " or "Self-monitoring…" mean? Each title on the figure should have a detail description at least in the supplementary data.

Regarding Figure 9, 10, the bars are not aligned with texts in the Y axis, which makes it difficult to read.

In the figures, the domain titles should be more identifiable. The authors should do more for the domain titles to be easily recognizable.

**Reviewer**	**2**
**Name**	**Gray, Emily**
**Affiliation**	**University of Otago, School of Physiotherapy**
**Date**	**08-Jun-2026**
**COI**	**None**

The systematic review addresses an important area of identifying the barriers and facilitators for attending cardiac rehabilitation in underserved populations. Incorporating evidence from both quantitative and qualitative research is a strength of this review. The introduction provides appropriate background information and justifies the rationale for the study aim. My comments are related mainly to the methodology for analysis and reporting of the findings to enhance readability and clarity.

It is good that the authors chose to map/interpret the findings in accordance with behaviour theory – however need to provide some justification for why the COM-B and TDF were selected as the behaviour change model of choice for this study.

The word 'indigenous' would be a highly relevant search term for a systematic review exploring perceptions of ethnic minorities; however, this was missing from search criteria. Could this have potentially led to some articles addressing cultural considerations being missed?

Inclusion criteria: please clarify how inclusion decisions were made regarding representation of underserved populations. Table 2 seems to indicate that studies that simply included participants from underserved populations within the wider study sample were included in the review. However, in Table 3 it is stated that 'Studies that did not focus on underserved or underrepresented groups' were excluded. Also, the classification of participants as "underserved" via inference based on study context needs some further detail regarding how these decisions were made. Overall, please clarify wording around how decisions for inclusion or exclusion were made regarding representation of underserved populations.

Results:

The one study from New Zealand has been reported in the paragraph reporting European based countries represented within the included articles. New Zealand is located in the south Pacific (east of Australia) and therefore is better placed in the paragraph above. The authors may be confused by the description of white/caucasian New Zealanders as 'NZ European'. This is in reference to ancestry resulting from colonisation and/or more recent immigration to New Zealand, rather than being actual Europeans.

Data analysis and reporting of results:

The reporting of the results is a bit disjointed and could be more integrated to improve readability. Suggest synthesizing results further into themes based on the COM-B headings that incorporate both barriers and facilitators. Also, it is not clear why different methods were used to analyze and synthesize the qualitative study findings and quantitative study findings into themes. Reporting would be greatly enhanced if the findings from both quantitative and qualitative data were synthesized into themes together under the COM-B headings. Merging the quantitative and qualitative data would also mean that the number of graphs could be condensed also. Integrating the data would also align with the following statement at the start of the methods section: "his mixed-methods systematic review integrating both qualitative and quantitative

evidence was undertaken..."

It is unclear which findings relate to which underserved populations and/or if all 'codes' within themes relate to all underserved populations. If possible, include reporting to highlight which barriers and facilitators (if any) relate specifically to women vs low socio-economic vs ethnic minorities.

The discussion mentions "A small number of qualitative studies also highlighted the positive influence of culturally aligned or faith-supported health beliefs" (pg 19, line 45) however this has not been reported in the written theme narratives in the results section. Cultural considerations is an important aspect of meeting the needs of ethnic minorities in health service delivery and therefore warrants inclusion in the written narrative of the themes in the results section. Also, the 'small number of studies' highlighting the influence of cultural

considerations could be due to not including ‘indigenous’ in the search strategy which may have led to potentially missing some relevant studies.

Figure 3:

- ‘Inappropriate CR equipment’ is listed under Physical Capability’ however would it fit better under ‘Physical Opportunity’ as this includes the TDF domain of ‘Environmental Context and Resources’.

- ‘Healthcare professionals own perception of physical activity or CR’ – would this fit better under ‘Social Opportunity’?

Discussion:

The rationale for singling out digital interventions as solutions for helping address motivational process related barriers in the discussion is unclear (pg 20 and 21). Suggest framing this section by saying that digital interventions are a way of addressing motivational and emotional related concerns in a way that overcomes previously mentioned barriers such as distance and employment and family commitments. Also, the promotion of use of digital interventions appears to be in conflict with the paragraph before which highlights digital exclusion as a barrier for those living with socioeconomic deprivation. While this is addressed in the section on clinical implications, I suggest moving commentary around addressing access to technology and technology literacy to earlier in the discussion.

It would be helpful for the reader to have more of a focus in the discussion on comparing and contrasting the current review findings in relation to wider literature. That is, how are the barriers and facilitators for underserved groups identified in this review similar and/or different to other cardiac rehabilitation participants.

VERSION 1 - AUTHOR RESPONSE

Reviewer 1

General Comments

Comment	Response
**the fact that they included so many different methodologies prevented them from performing quantitative analyses with some effect sizes or estimating at least the proportions of patients with the barriers or facilitators. Because the measurements frequently appeared in published studies often represent how easy to measure the variable rather than how important they are as determinants of a phenomenon, we should bear in mind that a large number of studies**	We agree that the frequency with which barriers and facilitators are reported in the literature does not necessarily reflect their relative importance or effect on cardiac rehabilitation participation. As this review synthesised evidence from diverse study designs, frequency counts were used to describe reporting patterns rather than estimate the magnitude of influence of individual factors. To address this concern, we have added clarifying statements in both the **Methods** (Data extraction and synthesis – pp. 9) and the **Strengths and limitations** sections (pp. 25) emphasising that

**regarding certain barriers/facilitators does not always mean their importance as factors in the clinical practices. For example, the long travel distance is the most frequently appeared barrier in the quantitative studies, but without the effect size of 1km travel distance increase to the CR initiation, the importance of the travel distance cannot be accurately estimated. Therefore, the authors should acknowledge the possibility that the plots might give some incorrect interpretation and impression to readers that the most important barrier is the travel distance and should try to clearly state this concern in the manuscript.**	frequency of reporting should not be interpreted as a measure of importance, causal influence, or effect size.
**Regarding Figure 6, "Side efect" is a spelling error.**	Figure 6 changed to **Figure 5** in the manuscript and has been corrected to read 'Side effects'.
**In the same figure, what does the "Specific… "or "Self-monitoring…" mean? Each title on the figure should have a detail description at least in the supplementary data.**	Figure 6 (**Figure 5**) has been revised to ensure that all labels are clearly displayed and readable in the revised manuscript.
**Regarding Figure 9, 10, the bars are not aligned with texts in the Y axis, which makes it difficult to read. In the figures, the domain titles should be more identifiable. The authors should do more for the domain titles to be easily recognizable.**	All figures have been revised to improve the visual distinction between the COM-B components and their corresponding TDF domains.

Reviewer 2

Comment	Response
**Introduction and Methods**	
**It is good that the authors chose to map/interpret the findings in accordance with behaviour theory – however need to provide some justification for why the COM-B and TDF were selected as the behaviour change model of choice for this study.**	These frameworks were chosen because they offer a comprehensive and theoretically informed approach to understanding behavioural determinants influencing cardiac rehabilitation participation and provide a structured framework for synthesising evidence across diverse study designs and underserved populations. The rationale for selecting the COM-B model and TDF added to the Introduction, Methods (Data extraction and data synthesis) and Discussion sections.
**The word 'indigenous' would be a highly relevant search term for a systematic review exploring**	We acknowledge that the specific term "Indigenous" was not included in the search strategy. As this review was international in scope, broader terms relating to

**perceptions of ethnic minorities; however, this was missing from search criteria. Could this have potentially led to some articles addressing cultural considerations being missed?**	ethnicity, minority populations, and underserved groups were used because terminology describing Indigenous populations varies between countries. We confirm that studies involving Aboriginal and Torres Strait Islander peoples in Australia and Māori populations in New Zealand were captured by the search strategy. The Strengths and limitations section has been revised to include this information. **(Strengths and limitations)**
**Inclusion criteria: please clarify how inclusion decisions were made regarding representation of underserved populations. Table 2 seems to indicate that studies that simply included participants from underserved populations within the wider study sample were included in the review. However, in Table 3 it is stated that 'Studies that did not focus on underserved or underrepresented groups' were excluded.**	The eligibility criteria have been clarified to specify that studies were eligible if they included participants from underserved populations either as the primary study population or as a subgroup within a broader cardiac rehabilitation sample. **(Methods – Study selection; Supplementary Table 2)**
**The classification of participants as "underserved" via inference based on study context needs some further detail regarding how these decisions were made. Overall, please clarify wording around how decisions for inclusion or exclusion were made regarding representation of underserved populations.**	The Methods section and Supplementary Table 3 have been expanded to provide additional detail regarding the identification of underserved populations when this was not explicitly reported. This includes the predefined criteria used to infer underserved status from participant characteristics and recruitment settings. **(Methods – Study selection; Supplementary Table 3)**
**Results**	
**The one study from New Zealand has been reported in the paragraph reporting European based countries represented within the included articles. New Zealand is located in the south Pacific (east of Australia) and therefore is better placed in the paragraph above. The authors may be confused by the description of white/Caucasian New Zealanders as 'NZ European'. This is in reference to ancestry resulting from colonisation and/or more recent immigration to New Zealand, rather than being actual Europeans.**	This was an error and has been corrected in the Results section on page 14.

**The reporting of the results is a bit disjointed and could be more integrated to improve readability.**	To improve readability and the overall flow of the Results section, we have restructured the presentation of findings to provide a more integrated narrative organised according to the COM-B model and corresponding TDF domains. **(Barriers and facilitators organised according to COM-B domains; pp 13-19)**
**Suggest synthesizing results further into themes based on the COM-B headings that incorporate both barriers and facilitators.**	The Results section has been reorganised so that qualitative and quantitative findings are presented together under the relevant COM-B components and corresponding TDF domains. Within each COM-B component, barriers and facilitators are described together where appropriate to provide a more integrated interpretation of the evidence.
**it is not clear why different methods were used to analyze and synthesize the qualitative study findings and quantitative study findings into themes.**	The Methods section has been expanded to clarify that qualitative and quantitative evidence required different approaches to because of their differing methodological characteristics. Qualitative findings were synthesised using thematic synthesis before deductive mapping to the COM-B model and TDF, whereas quantitative findings were summarised using vote counting because of substantial methodological heterogeneity that precluded meta-analysis.
**Reporting would be greatly enhanced if the findings from both quantitative and qualitative data were synthesized into themes together under the COM-B headings. Merging the quantitative and qualitative data would also mean that the number of graphs could be condensed also. Integrating the data would also align with the following statement at the start of the methods section: "his mixed-methods systematic review integrating both qualitative and quantitative evidence was undertaken..."**	We carefully considered condensing the graphical presentation by merging qualitative and quantitative findings as suggested by the Reviewer. However, because qualitative synthesis presents thematic findings whereas the quantitative synthesis summarises the frequency of reported barriers and facilitators, combining the figures would have reduced clarity and obscured the different types of evidence. Instead, we have reorganised the Results section under the COM-B components so that qualitative and quantitative findings are presented sequentially within each component, providing a more integrated narrative while retaining separate figures to preserve the clarity of the qualitative themes and quantitative frequency analyses.
**Integrating the data would also align with the following statement at the start of the methods section: "his mixed-methods systematic review integrating both qualitative and quantitative evidence was undertaken..."**	We have revised both the Methods and Results sections to improve consistency. The Results now reflect the mixed-methods design by integrating qualitative and quantitative evidence within the COM-B framework, while the Methods provide clearer justification for the different synthesis approaches used for each type of evidence. **(Methods; Results)**
**It is unclear which findings relate to which underserved populations and/or if all 'codes' within themes relate to all underserved populations. If possible, include reporting to highlight which barriers**	We have revised the Results and Discussion to clarify which barriers were more commonly reported by specific underserved populations. Although many barriers and facilitators were shared across groups, additional text now highlights those more frequently reported by women, ethnic minority groups, and

**and facilitators (if any) relate specifically to women vs low socio-economic vs ethnic minorities.**	individuals from lower socioeconomic backgrounds. **(Results; Discussion)**
**The discussion mentions "A small number of qualitative studies also highlighted the positive influence of culturally aligned or faith-supported health beliefs" (pg 19, line 45) however this has not been reported in the written theme narratives in the results section.**	The qualitative Results narrative has been expanded to describe how culturally aligned and faith-supported beliefs facilitated CR participation, rather than reporting these findings only as coded themes.
**Figure 3**	
**'Inappropriate CR equipment' is listed under Physical Capability' however would it fit better under 'Physical Opportunity' as this includes the TDF domain of 'Environmental Context and Resources'.**	We carefully reconsidered the coding of this theme. Although "Inappropriate CR equipment" could be interpreted as reflecting the environmental context, we chose to retain its classification within **Physical Capability (Skills)** because participants described difficulties using available equipment due to their physical condition and inability to comfortably perform the prescribed exercises, rather than lack of equipment or access to resources. We recognise that some COM-B and TDF domains may overlap, and this interpretation reflects the primary behavioural determinant identified from the participant narratives.
**Healthcare professionals' perceptions may fit better under Social Opportunity.**	We agree with the Reviewer. Following further review of the original studies, this theme reflected the influence of healthcare professionals' communication, encouragement, and support on patients' participation rather than healthcare professionals' knowledge. The theme has therefore been reclassified from Psychological Capability (Knowledge) to Social Opportunity (Social Influences) to better align with the COM-B model and TDF. The COM-B/TDF mapping, see **Figure 3** in the manuscript.
**Discussion**	
**The rationale for singling out digital interventions as solutions for helping address motivational process related barriers in the discussion is unclear (pg 20 and 21). Suggest framing this section by saying that digital interventions are a way of addressing motivational and emotional related concerns in a way that overcomes previously mentioned barriers such as distance and employment and family commitments.**	We agree that the role of digital interventions required clearer justification in relation to the barriers identified in this review. The Discussion has been revised to clarify that digital, home-based, and hybrid CR models are not presented solely as solutions for motivational barriers, but as approaches that can simultaneously address motivational and emotional concerns while overcoming practical barriers such as travel distance, transportation difficulties, employment, and caregiving responsibilities. The manuscript also emphasises that their effectiveness depends on equitable access to technology and adequate digital literacy. **(Discussion – Implications for practice)**

**The promotion of use of digital interventions appears to be in conflict with the paragraph before which highlights digital exclusion as a barrier for those living with socioeconomic deprivation. While this is addressed in the section on clinical implications, I suggest moving commentary around addressing access to technology and technology literacy to earlier in the discussion**	The Discussion has been revised to acknowledge earlier that digital exclusion, limited access to technology, and inadequate digital literacy may restrict the effectiveness of digital CR models among socioeconomically disadvantaged populations. We have clarified that digital, home-based, and hybrid interventions should be considered alongside strategies that address digital access and literacy to ensure equitable implementation.
**It would be helpful for the reader to have more of a focus in the discussion on comparing and contrasting the current review findings in relation to wider literature. That is, how are the barriers and facilitators for underserved groups identified in this review similar and/or different to other cardiac rehabilitation participants.**	The Discussion has been expanded to compare the findings of the present review with previous systematic reviews and the wider cardiac rehabilitation literature. It now highlights that several barriers identified in underserved populations, including travel distance, transportation difficulties, time constraints, comorbidities, and low referral rates, are consistent with those reported in the general CR population. Barriers that were more prominent among underserved groups, including cultural and language-related factors, caregiving responsibilities, financial constraints, and digital exclusion are also discussed, thereby emphasising the additional social and structural challenges experienced by these populations.

VERSION 2 - REVIEW

**Reviewer**	**2**
**Name**	**Gray, Emily**
**Affiliation**	**University of Otago, School of Physiotherapy**
**Date**	**18-Aug-2026**
**COI**	

Thank you for satisfactorily addressing all of my initial comments. There are a couple of minor typographical issues (for example in the introduction on page 6 it says '...compared to XXX.' - XXX needs to be replaced with text). Such changes can be made during the editorial process.

VERSION 2 - AUTHOR RESPONSE

Reviewer’s comments

Reviewer 2:
Thank you for satisfactorily addressing all of my initial comments. There are a couple of minor typographical issues (for example in the introduction on page 6 it says '...compared to XXX.' - XXX needs to be replaced with text). Such changes can be made during the editorial process.

Response:

This typographical issue was also highlighted by the Editor.

The missing text "XXX" in the Introduction has been added. The revised sentence on page 5 now reads:

"Ethnic minority groups also face barriers to CR participation, with evidence from the USA demonstrating substantially lower CR use among non-White patients compared with White patients (7.8% vs 19.6%)."
