# Supplementary material for: Barriers and facilitators of cardiac rehabilitation focusing on underserved groups: a mixed-methods systematic review
Source: BMJ Open. 2026 Oct 29;16(10):e119214. doi: 10.1136/bmjopen-2026-119214 (PMC13723823; doi:10.1136/bmjopen-2026-119214)
Supplement: Supplementary file 2 [file bmjopen-16-10-s001.docx]

**Supplementary Table 1:** Summary of database searches, search periods, and the number of records retrieved for the systematic review.

| Database searched | Coverage searched | Number of results |
| --- | --- | --- |
| MEDLINE (Ovid) | January 2000–September 2025 | 8,755 |
| PsycINFO (Ovid) | January 2000–September 2025 | 910 |
| CINAHL (EBSCO) | January 2000–September 2025 | 1,506 |
| PubMed | January 2000–September 2025 | 7,934 |
| Scopus | January 2000–September 2025 | 9,688 |
| Total | January 2000–September 2025 | 28,793 |

**Search strategy (Intervention AND Outcome AND cardiovascular)**

**MEDLINE (Ovid)**

1. exp Exercise/
2. exp Exercise Therapy/
3. Exercise Tolerance/
4. exp Sports/
5. Physical Exertion/
6. exp Physical Fitness/
7. exp "Physical Education and Training"/
8. exp Rehabilitation/
9. "Activities of Daily Living"/
10. cardiac rehabilitation/
11. exercis*.ti. or exercis*.ab.
12. sport*.ti. or sport*.ab.
13. (fitness or fitter or fit).ti. or (fitness or fitter or fit).ab.
14. (muscle* adj3 (train* or activ*)).ti. or (muscle* adj3 (train* or activ*)).ab.
15. (train* adj5 (strength* or aerobic* or exercise*)).ti. or (train* adj5 (strength* or aerobic* or exercise*)).ab.
16. ((aerobic or resistance) adj3 (train* or activ*)).ti. or ((aerobic or resistance) adj3 (train* or activ*)).ab.
17. (physical* adj5 (fit* or train* or therap* or activ* or strength or endur* or exert* or capacit*)).ti. or (physical* adj5 (fit* or train* or therap* or activ* or strength or endur* or exert* or capacit*)).ab.
18. ((exercise* or fitness) adj3 (treat* or interven* or program* or train* or physical or activ*)).ti. or ((exercise* or fitness) adj3 (treat* or interven* or program* or train* or physical or activ*)).ab.
19. (exercis* adj2 (toleran* or capacity)).ti. or (exercis* adj2 (toleran* or capacity)).ab.
20. rehabilitat*.ti. or rehabilitat*.ab.
21. walk*.ti. or walk*.ab.
22. ((lifestyle or life-style) adj5 activ*).ti. or ((lifestyle or life-style) adj5 activ*).ab.
23. ((lifestyle or life-style) adj5 physical*).ti. or ((lifestyle or life-style) adj5 physical*).ab.
24. (patient* adj5 educat*).ti. or (patient* adj5 educat*).ab
25. ((lifestyle or life-style) adj5 (interven* or program* or treatment*)).ti. or ((lifestyle or life-style) adj5 (interven* or program* or treatment*)).ab.
26. ((behavior* or behaviour*) adj5 (modify or modificat* or therap* or change)).ti. or ((behavior* or behaviour*) adj5 (modify or modificat* or therap* or change)).ab.
27. cardiac rehabilitation.ti. or cardiac rehabilitation.ab.
28. (gym or yoga or circuit training).ti. or (gym or yoga or circuit training).ab.
29. resistance training.ti. or resistance training.ab.
30. 1 OR 2 OR 3 OR 4 OR 5 OR 6 OR 7 OR 8 OR 9 OR 10 OR OR 11 OR 12 OR 13 OR 14 OR 15 OR 16 OR 17 OR 18 OR 19 OR 20 OR 21 OR 22 OR 23 OR 24 OR 25 OR 26 OR 17 OR 28 OR 29.
31. Increase participant*.ti. or Increase participant*.ab.
32. remain*.ti. or remain*.ab.
33. adhere*.ti. or adhere*.ab.
34. uptake.ti. or uptake.ab.
35. follow up.ti. or follow up.ab.
36. engage*.ti. or engage*.ab.
37. attend*.ti. or attend*.ab.
38. barrier*.ti. or barrier*.ab.
39. enable*.ti. or enable*.ab.
40. hinder*.ti. or hinder*.ab.
41. motivat*.ti. or motivat*.ab.
42. treatment adhere*.ti. or treatment adhere*.ab.
43. obstacle*.ti. or obstacle*.ab.
44. restrain*.ti. or restrain*.ab.
45. inhibit*.ti. or inhibit*.ab.
46. impede.ti. or impede.ab.
47. constrain.ti. or constrain.ab.
48. facilitator*.ti. or facilitator*.ab.
49. exp patient compliance/
50. exp "treatment adherence and compliance"/
51. (comply or compliance).ti. or (comply or compliance).ab.
52. 31 OR 32 OR 33 OR 34 OR 35 OR 36 OR 37 OR 38 OR 39 OR 40 OR 41 OR 42 OR 43 OR 44 OR 45 OR 46 OR 47 OR 48 OR 49 OR 50 OR 51.
53. exp Heart Diseases/
54. exp Coronary Disease/
55. Myocardial Infarction/
56. Heart Failure/
57. Myocardial Ischemia/
58. Percutaneous Coronary Intervention/
59. Angioplasty/
60. (cabg or coronary artery bypass graft).ti. or (cabg or coronary artery bypass graft).ab.
61. (heart disease* or Coronary Disease*).ti. or (heart disease* or Coronary Disease*).ab.
62. (heart infarc* or myocardial infarction).ti. or (heart infarc* or myocardial infarction).ab.
63. (PCI or Percutaneous Coronary Intervention).ti. or (PCI or Percutaneous Coronary Intervention).ab.
64. angioplast*.ti. or angioplast*.ab.
65. stent*.ti. or stent*.ab.
66. ptca.ti. or ptca.ab.
67. heart problem*.ti. or heart problem*.ab.
68. cardiovascular.ti. or cardiovascular.ab.
69. 53 OR 54 OR 55 OR 56 OR 57 OR 58 OR 59 OR 60 OR 61 OR 62 OR 63 OR 64 OR 65 OR 66 OR 67 OR 68.
70. Underrepresented.ti. or Underrepresented.ab.
71. Equity.ti. or Equity.ab.
72. Underserved.ti. or Underserved.ab.
73. Minority.ti. or Minority.ab.
74. Socioeconomic.ti. or Socioeconomic.ab.
75. Ethnic.ti. or Ethnic.ab.
76. (Diverse or Diversity).ti. or (Diverse or Diversity).ab.
77. low-income status.ti. or low-income status.ab.
78. exp Health Equity/
79. exp Minority Groups/
80. *"Ethnic and Racial Minorities"/
81. 70 OR 71 OR 72 OR 73 OR 74 OR 75 OR 76 OR 77 OR 78 OR 79 OR 80.
82. limit 84 to (full text and humans and yr="2000 - 2025" and "all adult (19 plus years)")

**CINAHL (EBSCO)**

S1 MH exercise

S2 MH "Exercise Therapy"

S3 MH "Exercise Tolerance"

S4 MH Sports

S5 MH "Physical Activity"

S6 MH "Physical Fitness"

S7 MH "Physical Education and Training"

S8 MH "Rehabilitation"

S9 MH "Activities of Daily Living"

S10 MH "Cardiac Rehabilitation"

S11 TI exercis* OR AB exercis*

S12 TI sport* OR AB sport*

S13 TI ( (fitness or fitter or fit) ) OR AB ( (fitness or fitter or fit) )

S14 TI ( (muscle* N3 (train* or activ*)) ) OR AB ( (muscle* N3 (train* or activ*)) )

S15 TI ( (train* N5 (strength* or aerobic* or exercise*)) ) OR AB ( (train* N5 (strength* or aerobic* or exercise*)) )

S16 TI ( ((aerobic or resistance) N3 (train* or activ*)) ) OR AB ( ((aerobic or resistance) N3 (train* or activ*)) )

S17 TI ( (physical* N5 (fit* or train* or therap* or activ* or strength or endur* or exert* or capacit*)) ) OR AB ( (physical* N5 (fit* or train* or therap* or activ* or strength or endur* or exert* or capacit*)) )

S18 TI ( ((exercise* or fitness) N3 (treat* or interven* or program* or train* or physical or activ*)) ) OR AB ( ((exercise* or fitness) N3 (treat* or interven* or program* or train* or physical or activ*)) )

S19 TI ( (exercis* N2 (toleran* or capacity)) ) OR AB ( (exercis* N2 (toleran* or capacity)) )

S20 TI rehabilitat* OR AB rehabilitat*

S21 TI walk* OR AB walk*

S22 TI ( ((lifestyle or life-style) N5 (activ* or physical*)) ) OR AB ( ((lifestyle or life-style) N5 (activ* or physical*)) )

S23 TI ( ((lifestyle or life-style) N5 (interven* or program* or treatment*)) ) OR AB ( ((lifestyle or life-style) N5 (interven* or program* or treatment*)) )

S24 TI ( ((behavior* or behaviour*) N5 (modify or modificat* or therap* or change)) ) OR AB ( ((behavior* or behaviour*) N5 (modify or modificat* or therap* or change)) )

S25 TI "cardiac rehabilitation" OR AB "cardiac rehabilitation"

S27 TI resistance training OR AB resistance training

S28 TI ( (gym or yoga or circuit training) ) OR AB ( (gym or yoga or circuit training) )

S29 S1 OR S2 OR S3 OR S4 OR S5 OR S6 OR S7 OR S8 OR S9 OR S10 OR S11 OR S12 OR S13 OR S14 OR S15 OR S16 OR S17 OR S18 OR S19 OR S20 OR S21 OR S22 OR S23 OR S24 OR S25 OR S26 OR S27 OR S28.

S30 TI "Increase participants" OR AB "Increase participants"

S31 remain*

S32 adhere*

S33 uptake

S34 "follow up"

S35 engage*

S36 attend*

S37 barrier*

S38 enable*

S39 hinder*

S40 motivat*

S41 "treatment adhere*"

S42 obstacle*

S43 restrain*

S44 inhibit*

S45 impede

S46 constrain

S47 motivate*

S48 S30 OR S31 OR S32 OR S33 OR S34 OR S35 OR S36 OR S37 OR S38 OR S39 OR S40 OR S41 OR S42 OR S43 OR S44 OR S45 OR S46 OR S47

S49 S29 AND S48

S50 MH Heart Diseases

S51 MH Coronary Disease

S52 MH Myocardial Infarction

S53 cabg

S54 coronary artery bypass graft

S55 MH Heart Failure

S56 MH Myocardial Ischemia

S57 MH Percutaneous Coronary Intervention

S58 MH Angioplasty

S59 heart disease*

S60 "Coronary Disease"

S61 "heart infarc*"

S62 myocardial infarction

S63 CABG OR PCI

S64 angioplast*

S65 stent*

S66 ptca

S67 S50 OR S51 OR S52 OR S53 OR S54 OR S55 OR S56 OR S57 OR S58 OR S59 OR S60 OR S61 OR S62 OR S63 OR S64 OR S65 OR S66

S68 S49 AND S67

S69 Full-Text Online; Publication Year: 2000-2025; Human; Age Groups: All Adult.

**APA PsycINFO (OVID)**

S1 MA Sports

S2 MA exercise

S3 MA Physical Exertion

S4 MA Physical Fitness

S5 MA ("Physical Education and Training")

S6 MA Exercise Tolerance

S7 MA Rehabilitation

S8 MA "Activities of Daily Living"

S9 MA Rehabilitation Centers

S10 TI exercis* OR AB exercis*

S11 TI sport* OR AB sport*

S12 TI ( (fitness or fitter or fit) ) OR AB ( (fitness or fitter or fit) )

S13 TI rehabilitat* OR AB rehabilitat*

S14 TI walk* OR AB walk*

S15 TI ( (("lifestyle" or life-style) N2 activ*) ) OR AB ( (("lifestyle" or life-style) N2 activ*) )

S16 TI ( (muscle* N3 (train* or activ*)) ) OR AB ( (muscle* N3 (train* or activ*)) )

S17 TI ( (train* N5 (strength* or aerobic* or exercise*)) ) OR AB ( (train* N5 (strength* or aerobic* or exercise*)) )

S18 TI ( ((aerobic or resistance) N3 (train* or activ*)) ) OR AB ( ((aerobic or resistance) N3 (train* or activ*)) )

S19 TI ( (physical* N5 (fit* or train* or therap* or activ* or strength or endur* or exert* or capacit*)) ) OR AB ( (physical* N5 (fit* or train* or therap* or activ* or strength or endur* or exert* or capacit*)) )

S20 TI ( ((exercise* or fitness) N3 (treat* or interven* or program* or train* or physical or activ*)) ) OR AB ( ((exercise* or fitness) N3 (treat* or interven* or program* or train* or physical or activ*)) )

S21 TI ( exercis* N2 (toleran* or capacity) ) OR AB ( exercis* N2 (toleran* or capacity) )

S22 TI ( ("lifestyle" or life-style) N5 physical* ) OR AB ( ("lifestyle" or life-style) N5 physical* )

S23 TI ( (lifestyle or life-style) N5 (interven* or program* or treatment*) ) OR AB ( (lifestyle or life-style) N5 (interven* or program* or treatment*) )

S24 TI ( (behavior* or behaviour*) N5 (modify or modificat* or therap* or change) ) OR AB ( (behavior* or behaviour*) N5 (modify or modificat* or therap* or change) )

S25 TI ( motivat* N5 (intervention or interv*) ) OR AB ( motivat* N5 (intervention or interv*) )

S26 TI home based OR AB home based

S27 TI centre based OR AB center based

S28 S1 OR S2 OR S3 OR S4 OR S5 OR S6 OR S7 OR S8 OR S9 OR S10 OR S11 OR S12 OR S13 OR S14 OR S15 OR S16 OR S17 OR S18 OR S19 OR S20 OR S21 OR S22 OR S23 OR S24 OR S25 OR S26 OR S27

S29 TI Increase participant* OR AB Increase participant*

S30 TI remain* OR AB remain*

S31 TI adhere* OR AB adhere*

S32 TI uptake OR AB uptake

S33 TI "follow up" OR AB "follow up"

S34 TI engage* OR AB engage*

S35 TI attend* OR AB attend*

S36 TI barrier* OR AB barrier*

S37 TI enable* OR AB enable*

S38 TI hinder* OR AB hinder*

S39 TI ( motivat* N5 (intervention or interv*) ) OR AB ( motivat* N5 (intervention or interv*) )

S40 TI treatment adhere* OR AB treatment adhere*

S41 TI obstacle* OR AB obstacle*

S42 TI restrain* OR AB restrain*

S43 TI inhibit* OR AB inhibit*

S44 TI impede OR AB impede

S45 TI constrain OR AB constrain

S46 S29 OR S30 OR S31 OR S32 OR S33 OR S34 OR S35 OR S36 OR S37 OR S38 OR S39 OR S40 OR S41 OR S42 OR S43 OR S44 OR S45

S47 S28 AND S46

S48 MA Heart Diseases

S49 MA Coronary Disease

S50 MA Myocardial Infarction

S51 MA Heart Failure

S52 MA Myocardial Ischemia

S53 MA Percutaneous Coronary Intervention

S54 MA Angioplasty

S55 TI ( cabg or coronary artery bypass graft or valve surgery or heart surgery or cardiac surgery ) OR AB ( cabg or coronary artery bypass graft or valve surgery or heart surgery or cardiac surgery )

S56 TI heart disease* OR AB heart disease*

S57 TI Coronary N2 Disease* OR AB Coronary N2 Disease*

S58 TI heart infarc* OR AB heart infarc*

S59 TI myocardial infarction OR AB myocardial infarction

S60 TI ( pci or percutaneous coronary intervention or angioplasty ) OR AB ( pci or percutaneous coronary intervention or angioplasty )

S61 TI angioplast* OR AB angioplast*

S62 TI stent* OR AB stent*

S63 TI ptca OR AB ptca

S64 TI ( heart disease or cardiovascular disease or coronary artery disease or heart health ) OR AB ( heart disease or cardiovascular disease or coronary artery disease or heart health )

S65 TI heart problems OR AB heart problems

S66 S48 OR S49 OR S50 OR S51 OR S52 OR S53 OR S54 OR S55 OR S56 OR S57 OR S58 OR S59 OR S60 OR S61 OR S62 OR S63 OR S64 OR S65

S67 S47 AND S66

S68 Full-Text Online; Publication Year: 2000-2025; Age Groups: Adulthood (18 yrs & older); Population Group: Human

**Scopus**

1. TITLE-ABS-KEY ( sport* )
2. TITLE-ABS-KEY ( "Physical Exertion" )
3. TITLE-ABS-KEY ( exercise* )
4. TITLE-ABS-KEY ( "Physical Fitness" ) )
5. TITLE-ABS-KEY ( "Physical Education and Training" )
6. TITLE-ABS-KEY ( "Exercise Tolerance" )
7. TITLE-ABS-KEY ( rehabilitat* ) )
8. TITLE-ABS-KEY ( "Activities of Daily Living" )
9. TITLE-ABS-KEY ( "Rehabilitation Centers" )
10. TITLE-ABS-KEY ( ( fitness OR fitter OR fit ) ) )
11. TITLE-ABS-KEY ( walk* )
12. TITLE-ABS-KEY ( ( ( "lifestyle" OR life-style ) W/2 activ* ) )
13. TITLE-ABS-KEY ( ( muscle* W/3 ( train* OR activ* ) ) ) )
14. TITLE-ABS-KEY ( ( train* W/5 ( strength* OR aerobic* OR exercise* ) ) )
15. TITLE-ABS-KEY ( ( ( aerobic OR resistance ) W/3 ( train* OR activ* ) ) )
16. TITLE-ABS-KEY ( ( physical* W/5 ( fit* OR train* OR therap* OR activ* OR strength OR endur* OR exert* OR capacit* ) ) ) )
17. TITLE-ABS-KEY ( ( ( exercise* OR fitness ) W/3 ( treat* OR interven* OR program* OR train* OR physical OR activ* ) ) )
18. TITLE-ABS-KEY ( ( exercis* W/2 ( toleran* OR capacity ) ) )
19. TITLE-ABS-KEY ( ( ( "lifestyle" OR life-style ) W/5 physical* ) ) )
20. TITLE-ABS-KEY ( ( ( lifestyle OR life-style ) W/5 ( interven* OR program* OR treatment* ) ) )
21. TITLE-ABS-KEY ( ( ( behavior* OR behaviour* ) W/5 ( modify OR modificat* OR therap* OR change ) ) )
22. TITLE-ABS-KEY ( ( motivat* W/5 ( intervention OR interv* ) ) ) )
23. TITLE-ABS-KEY ( "home based" )
24. TITLE-ABS-KEY ( "center based" )
25. TITLE-ABS-KEY ( "centre based" ) )
26. 1 OR 2 OR 3 OR 4 OR 5 OR 6 OR 7 OR 8 OR 9 OR 10 OR 11 OR 12 OR 13 OR 14 OR 15 OR 16 OR 17 OR 18 OR 19 OR 20 OR 21 OR 22 OR 23 OR 24 OR 25
27. TITLE-ABS-KEY ( "Increase participants" )
28. TITLE-ABS-KEY ( remain* )
29. TITLE-ABS-KEY ( adhere* ) )
30. TITLE-ABS-KEY ( uptake )
31. TITLE-ABS-KEY ( "follow up" )
32. TITLE-ABS-KEY ( engage* ) )
33. TITLE-ABS-KEY ( attend* )
34. TITLE-ABS-KEY ( barrier* )
35. TITLE-ABS-KEY ( enable* ) )
36. TITLE-ABS-KEY ( hinder* )
37. TITLE-ABS-KEY ( ( motivat* W/5 ( intervention OR interv* ) ) )
38. TITLE-ABS-KEY ( "treatment adhere* " ) )
39. TITLE-ABS-KEY ( obstacle* )
40. TITLE-ABS-KEY ( restrain* )
41. TITLE-ABS-KEY ( inhibit* ) )
42. TITLE-ABS-KEY ( impede )
43. TITLE-ABS-KEY ( constrain ) )
44. 27 OR 28 0R 29 OR 30 OR 31 OR 32 OR 33 OR 34 OR 35 OR 36 OR 37 OR 38 OR 39 OR 40 OR 41 OR 42 OR 43
45. 26 AND 44
46. TITLE-ABS-KEY ( "Heart Diseases" OR "Heart disease" )
47. TITLE-ABS-KEY ( "Coronary Disease" OR "Coronary Diseases" )
48. TITLE-ABS-KEY ( "Myocardial Infarction" )
49. TITLE-ABS-KEY ( "Heart Failure" )
50. TITLE-ABS-KEY ( "Myocardial Ischemia" )
51. TITLE-ABS-KEY ( "Percutaneous Coronary Intervention" )
52. TITLE-ABS-KEY ( angioplasty )
53. TITLE-ABS-KEY ( cabg OR "coronary artery bypass graft" OR "valve surgery" OR "heart surgery" OR "cardiac surgery" )
54. TITLE-ABS-KEY ( "heart infarc*" )
55. TITLE-ABS-KEY ( pci OR "percutaneous coronary intervention" )
56. TITLE-ABS-KEY ( stent* )
57. TITLE-ABS-KEY ( ptca )
58. 46 OR 47 OR 48 OR 49 OR 50 OR 51 OR 52 OR 53 OR 54 OR 55 OR 56 OR 57
59. 45 AND 58
60. Full-Text Online; Publication Year: 2000-2025; Age Groups: Adulthood (18 yrs & older); Population Group: Human

**PubMed**

**((((((((((((((((((Coronary Disease*[MeSH Terms]) OR (Heart Disease*[MeSH Terms])) OR (Heart Disease*[MeSH Terms])) OR (Myocardial Infarction[MeSH Terms])) OR (Heart Failure[MeSH Terms])) OR (Myocardial Ischemia[MeSH Terms])) OR (Percutaneous Coronary Intervention[MeSH Terms])) OR (Angioplasty[MeSH Terms])) OR (cabg[Title/Abstract] OR coronary artery bypass graft[Title/Abstract])) OR (heart disease*[Title/Abstract] OR Coronary Disease*[Title/Abstract])) OR (heart infarc*[Title/Abstract] OR myocardial infarction[Title/Abstract])) OR (PCI[Title/Abstract] OR Percutaneous Coronary Intervention[Title/Abstract])) OR (angioplast*[Title/Abstract])) OR (stent*[Title/Abstract] OR ptca[Title/Abstract])) OR (heart problem*[Title/Abstract])) OR (cardiovascular[Title/Abstract])) AND ((classicalarticle[Filter] OR clinicaltrial[Filter] OR comparativestudy[Filter] OR controlledclinicaltrial[Filter] OR interview[Filter] OR meta-analysis[Filter] OR observationalstudy[Filter] OR randomizedcontrolledtrial[Filter] OR review[Filter] OR systematicreview[Filter]) AND (fft[Filter]) AND (humans[Filter]) AND (2000:2024[pdat]) AND (alladult[Filter]))) AND (((((((((((((((((((((((comply[Title/Abstract] OR compliance[Title/Abstract]) OR (treatment adherence and compliance[MeSH Terms])) OR (patient compliance[MeSH Major Topic])) OR (patient compliance[MeSH Terms])) OR (facilitator*[Title/Abstract])) OR (constrain[Title/Abstract])) OR (impede[Title/Abstract])) OR (inhibit*[Title/Abstract])) OR (restrain*[Title/Abstract])) OR (obstacle*[Title/Abstract])) OR (treatment adhere*[Title/Abstract])) OR (motivat*[Title/Abstract])) OR (hinder[Title/Abstract])) OR (enable*[Title/Abstract])) OR (barrier*[Title/Abstract])) OR (engage*[Title/Abstract])) OR (attend*[Title/Abstract])) OR (follow up[Title/Abstract])) OR (uptake[Title/Abstract])) OR (adhere*[Title/Abstract])) OR (remain*[Title/Abstract])) OR (Increase participant*.[Title/Abstract]))) AND ((classicalarticle[Filter] OR clinicaltrial[Filter] OR comparativestudy[Filter] OR controlledclinicaltrial[Filter] OR interview[Filter] OR meta-analysis[Filter] OR observationalstudy[Filter] OR randomizedcontrolledtrial[Filter] OR review[Filter] OR systematicreview[Filter]) AND (fft[Filter]) AND (humans[Filter]) AND (2000:2024[pdat]) AND (alladult[Filter])))) AND ((((((((((((((((((((((exercise[MeSH Terms]) OR (Exercise Therapy[MeSH Terms])) OR (physical therapy[MeSH Terms])) OR (Exercise Tolerance[MeSH Terms])) OR (Sports[MeSH Terms])) OR (Physical Exertion[MeSH Terms])) OR (Physical Fitness[MeSH Terms])) OR (Physical Education and Training[MeSH Terms])) OR (Rehabilitation[MeSH Terms])) OR ("Activities of Daily Living"[MeSH Terms])) OR (Cardiac rehabilitation[MeSH Terms])) OR (exercis*[Title/Abstract])) OR (sport*[Title/Abstract])) OR ((fitness[Title/Abstract] OR fitter[Title/Abstract] OR fit[Title/Abstract]))) OR (physical fit*[Title/Abstract] OR physical train*[Title/Abstract] OR physical therap*[Title/Abstract] OR physical activ*[Title/Abstract] OR physical strength[Title/Abstract] OR physical endur*[Title/Abstract] OR physical exert*[Title/Abstract] OR physical capacit*[Title/Abstract])) OR (exercise* treat*[Title/Abstract] OR exercise* interven*[Title/Abstract] OR exercise* program*[Title/Abstract] OR exercise* train*[Title/Abstract] OR physical exercise*[Title/Abstract] OR exercise* activ*[Title/Abstract])) OR (rehabilitat*[Title/Abstract])) OR (patient* educat*[Title/Abstract])) OR ((behaviour* modify[Title/Abstract] OR behaviour* modificat*[Title/Abstract] OR behaviour* therap*[Title/Abstract] OR behaviour* change[Title/Abstract]))) OR (resistance training[Title/Abstract]) AND (fft[Filter]) AND (humans[Filter]) AND (2000:2024[pdat]) AND (alladult[Filter])))** Filters: **Full text, Humans, Adult: 19+ years, from 2000 – 2025**

**Supplementary Table 2:** Eligibility criteria for the systematic review

| Inclusion criteria | Exclusion criteria |
| --- | --- |
| • Adults (≥18 years old) diagnosed with any form of heart disease, where the study sample included individuals from underserved populations (e.g., ethnic minority groups, women, or those from lower socioeconomic backgrounds), either as the entire study population or as a subgroup within a broader sample. | **•** Focus exclusively on White patients or individuals with high SES. |
| • Qualitative, Quantitative or mixed methods evidence available in Full text | **•** Evaluate interventions that are not comparable or relevant to the scope of the review. |
| • Barriers to, or facilitators of participation or completing CR (one of the main outcomes of the study) | • Published as conference abstracts, case studies, or editorial articles |

**Supplementary Table 3:** Definitions and use of terms describing inclusion of underserved groups.

| Terms | Definition / Criteria | Example / Explanation |
| --- | --- | --- |
| Reported | The study provided descriptive demographic information about the characteristics (e.g., ethnicity, income, education level, gender composition) of underserved participants. | The paper stated that “30% of participants were from ethnic minority backgrounds” or “half of the sample had low income.” |
| Included | The study explicitly stated that individuals from underserved populations were part of the sample, even if no exact demographic proportions were reported. This also included studies where underserved status could be reasonably inferred from participant characteristics or recruitment settings (e.g., low-income communities, socioeconomically deprived areas, publicly funded healthcare settings, or predominantly ethnically diverse populations). | A study noting that “participants were recruited from a low-income community” or that the research took place in a predominantly ethnically diverse area, even if specific data were not disaggregated. |
| Not stated | The study did not mention any participant characteristics related to underserved status. | No reference to ethnicity, socioeconomic status, or related demographic information in the sample description. |

**Supplementary Table 4:** Reasons for exclusion at the full-text screening stage

| **Reason for exclusion** | **Description** | **Number of studies (n=65)** |
| --- | --- | --- |
| Wrong population | Studies that did not include patients with cardiovascular disease or did not include participants from underserved or under-represented groups and did not report data relevant to these populations. (e.g., healthy adults, other chronic diseases). | 34 |
| Wrong outcome | Studies that did not report barriers, facilitators, or determinants of CR participation | 25 |
| Wrong publication type | Editorials, commentaries, conference abstracts, or case reports that did not provide sufficient methodological or results information. | 4 |
| Wrong intervention | Studies that evaluated interventions other than CR (e.g., pharmacological, or surgical interventions). | 2 |

**Supplementary Table 5:** Characteristics of the 85 included studies and the main barriers and facilitators to cardiac rehabilitation participation and adherence.

| Study | Country | Study design | Sample size (M/F) | The Underserved group(s) included | Main barriers/facilitators relevant to this review |
| --- | --- | --- | --- | --- | --- |
| Allen et al., 2004 | USA | Prospective longitudinal cohort study | 253 Females | Women included; ethnic minority participants included (African American); low educational attainment represented; low SES represented. | Barriers: low income, African American ethnicity, low education, comorbidities and lack of referral. Facilitators: physician referral, family support and encouragement from healthcare professionals. |
| Alsaleh & Baniyasin, 2023 | Jordan | Cross-sectional study | 400 (259/141) | Women included; low educational attainment represented; low SES represented | Barriers: anxiety, lack of time, low motivation, bad weather and discomfort during physical activity. Facilitators: perceived health benefits, improved fitness and psychological well-being. |
| Amirova et al., 2022 | UK | Qualitative interviews (TDF) | 16 (12/4) | Predominantly White British; ethnic minority participants included (British Pakistani and Asian Other); educational attainment reported; SES not reported. | Barriers: concerns about physical activity, symptoms, low confidence. Facilitators: social support, goal setting, action planning. |
| Andraos et al., 2015 | Canada | Randomised controlled trial with mixed-methods evaluation | 169 Females | Women included; ethnic minority participants included. | Barriers: time conflicts, transport and preference mismatch with allocated programme. Facilitators: supervised programmes, women-only environment, programme flexibility and patient preference. |
| Antoniou et al., 2023 | Greece | Cross-sectional psychometric validation study | 110 (97/13) | Women included; low educational attainment represented; low SES represented. | Barriers: distance to CR centre, cost, lack of information about CR, already exercising at home. |
| Astin et al., 2008 | UK | Comparative qualitative interviews | 65 (36/29) | South Asian (Pakistani Muslim, Indian Sikh, Indian Hindu) and White-European participants. | Barriers: language, communication, misconceptions. Facilitators: family support, culturally responsive communication. |
| Bakhshayeh et al., 2021 | Iran | Cross-sectional study | 204 (145/59) | Women included; low SES represented. | Barriers: transportation, travel cost, distance, lack of insurance coverage, poor physician referral/knowledge. Facilitators: preference for home-based CR with specialist support, education and periodic follow-up. |
| Banerjee et al., 2010 | Canada | Qualitative descriptive study | 16 (13/3) | South Asian ethnicity (Indian, Pakistani, Bangladeshi and Sri Lankan origin); immigrant population. | Facilitators: supervised CR, physician/family support, culturally sensitive care. |
| Beleigoli et al., 2024 | Australia | Mixed methods (retrospective cohort + qualitative interviews/focus groups) | Quantitative: 16,159 (9,549/6,610); Qualitative: 28 (22/6) | Women included; low SES; rural populations. | Barriers: transport and travel, costs, inflexible scheduling, limited social support, poor communication during transition of care, long waiting times, lack of integrated/person-centred care, inadequate health literacy support. Facilitators: telehealth delivery, flexible scheduling, family support, active follow-up, tailored education and exercise, integrated care. |
| Brual et al., 2010 | Canada | Prospective cohort study | 1268 (910/358) | Women included; ethnic minority participants included; SES reported; Rural/remote populations (geographical disadvantage). | Barrier: travel time/geographical accessibility. Patients living ≥60 minutes from a CR programme were significantly less likely to be referred to and enroll, although participation after enrolment was unaffected. |
| Brual et al., 2012 | Canada | Secondary analysis of a prospective cohort | 373 (274/99) | Women included; ethnic minority participants included; SES reported; Rural/remote populations (geographical disadvantage). | Barrier: geographical access to CR. Facilitator: home-based CR for lower-risk patients. |
| Bryant et al., 2021 | USA | Observational quality improvement study | 673 (437/236) | Women included; ethnic minority participants included | Barrier: geographical access to CR. Facilitator: home-based CR for lower-risk patients. |
| Cao et al., 2021 | China | Prospective longitudinal study | 283 (164/119) | Women included; rural residents represented; low SES reported | Barriers: older age, poor CAD knowledge and low self-management. Facilitators: shared decision-making, better CAD knowledge and self-management. |
| Chauhan et al., 2010 | England | Qualitative study using semi-structured face-to-face interviews (framework analysis) | 20 (13/7) | South Asian ethnicity (Pakistani, Indian and Bangladeshi); multilingual population. | Barriers: poor understanding of CR, communication and language barriers, cultural/religious factors, transport and programme timing. Facilitators: family and healthcare professional support, social networks and culturally appropriate care. |
| Chong et al., 2024 | Malaysia | Cross-sectional study | 240 (194/46) | Women included; ethnic minority participants included; low educational attainment represented; low SES represented. | Barriers: perceived lack of need, logistical barriers (transport, distance, family responsibilities), self-driving, and ethnicity. Facilitators: social support, management of comorbidities, acceptance of technology, and home-/hybrid CR. |
| Cossette et al., 2012 | Canada | Randomised controlled trial | 242 (194/48) | Women included; educational attainment reported | Barriers: perceived barriers to CR, low perceived personal control and limited professional endorsement. Facilitators: tailored nursing intervention, education, family support and personalised follow-up, resulting in increased CR enrolment. |
| Damery et al., 2025 | England | Qualitative study (focus groups and semi-structured interviews) | 6 Males | Ethnic minority participants included; SES reported | Barriers: technology, governance, training. Facilitators: convenience, tailoring, remote monitoring. |
| Dankner et al., 2015 | Israel | Multicentre controlled interventional cohort study | 1,024 (780/244) | Women included; ethnic minority participants include; low educational attainment represented; low SES represented | Barriers: lack of knowledge about CR, low income, lower education and female sex. Facilitators: staff education, patient education, written and verbal information, telephone follow-up and professional healthcare recommendation. |
| Donnelly et al., 2012 | Qatar | In-depth interviews | 50 Females | Women; educational attainment reported; SES reported. | Barriers: cultural norms, family responsibilities, climate and health limitations. Facilitators: family support, healthcare support, religion and motivation for healthy lifestyles. |
| Dunlay et al., 2009 | USA | Prospective cohort study | 179 (118/61) | Women included; ethnic minority reported; lower educational attainment represented. | Barriers: older age, female sex, diabetes, previous MI, previous CR attendance, lack of in-hospital referral, lower education, inability to drive, low perceived need for CR, lower perceived importance of CR. Facilitators: in-hospital referral, cardiologist involvement, reperfusion therapy, better perceived health, ability to drive, post-secondary education, high perceived necessity and importance of CR. |
| Endo et al., 2015 | Japan | Single-centre case-control study | 257 (162/95) | Women included; Rural/remote populations (geographical access) | Barriers: older age and greater distance from the hospital reduced outpatient CR enrolment. Facilitators: younger age, shorter travel distance, history of ischaemic heart disease and prescription of antidepressants/sleeping medication were associated with better adherence. |
| Evangelista et al., 2003 | USA | Comparative cross-sectional study | 140 (94/46) | Women included; ethnic minority participants include; low educational attainment represented; low SES represented; | Barriers: difficulty adhering to exercise and dietary recommendations, lack of transportation, travel distance, treatment costs, forgetfulness, medication side effects, poor motivation, fatigue, pain and dizziness. Facilitators: patient education, counselling, social support and strategies to reduce perceived difficulty with treatment adherence. |
| Field et al., 2022 | Australia | Qualitative descriptive case study using semi-structured interviews | 44 (26/18) | Rural and remote populations; Aboriginal and Torres Strait Islander participants included; women included; low educational attainment and socioeconomic disadvantage reported. | Barriers: poor referral pathways, limited CR availability, poor understanding of CR, geographic distance and limited multidisciplinary care. Facilitators: local health services, improved referral pathways, home-based support and multidisciplinary care. |
| Frost et al., 2019 | UK | Mixed methods process evaluation alongside a multicentre RCT | Quantitative: 107 intervention patients (26 women, 81 men); 53 caregivers. Qualitative: 19 patients; 17 caregivers | Women included; ethnic minority reported; SES reported | Barriers: multimorbidity, limited social and material support, low intervention fidelity, poor facilitator–patient fit, dependence on facilitators, and inadequate caregiver involvement. Facilitators: tailored intervention delivery, skilled facilitation, caregiver and family support, self-monitoring, personalised education, behaviour-changing techniques and establishing shared understanding and goals. |
| Gaalema et al., 2024 | USA | Randomised controlled trial | 192 (125/67) | Women included; ethnic minority reported; low SES; low educational attainment represented. | Barriers: financial hardship, executive function challenges and difficulty navigating healthcare. Facilitators: financial incentives, case management, needs assessment and ongoing support improved CR adherence. |
| Galdas & Kang, 2010 | Canada | Interpretive qualitative study using in-depth interviews | 15 (10/5) | Ethnic minority (Punjabi Sikh); women included. | Barriers: transport difficulties, language barriers and limited culturally appropriate advice. Facilitators: culturally tailored dietary advice, peer support, multidisciplinary support and ongoing dialogue with healthcare professionals. |
| Galdas et al., 2012 | Canada | Qualitative interpretive descriptive study | 15 (10/5) | Ethnic minority (Punjabi Sikh); women included. | Barriers: uncertainty about safe exercise intensity, fatigue and weakness, preference for informal exercise (walking), migration-related challenges, unfamiliarity with gym settings, language barriers. Facilitators: medically supervised exercise, culturally appropriate advice, walking as an acceptable form of physical activity. |
| Ghisi et al., 2013 | Brazil | Cross-sectional comparative study | 628 (271/357) | Women included; low SES; rural populations. | Barriers: lack of CR awareness, distance, cost, transport, work and family responsibilities, and limited access. Facilitators: free CR programmes, improved CR availability and reducing access barriers. |
| Ghisi et al., 2023 | International (16 countries) | Cross-sectional international | 2163 (1247/916) | Women; ethnic minority participants included; educational attainment reported; low SES represented. | Barriers: lack of CR awareness, lack of programme contact, cost, distance, transport and family responsibilities. Facilitators: targeted barrier mitigation strategies, improved communication, and tailored support for women. |
| Grace et al., 2005 | Canada | Prospective controlled intervention study | 80 Females | Women included; ethnic minority participants included; low educational attainment represented; low SES represented. | Barriers: low physician referral, lack of discussion about CR, travel/transport, lack of physician encouragement, time constraints, comorbidities, preference for self-recovery. Facilitators: psychoeducational brochure, motivational interview, patient-initiated discussion with healthcare providers, increased referral discussions. |
| Grace et al., 2009 | Canada | Prospective multicentre survey (secondary analysis) | 1273 (843/430) | Women included; ethnic minority participants included; low educational attainment represented; SES reported. | Barriers: lack of CR awareness, lack of physician encouragement, comorbidities, perception that exercise is tiring/painful, confidence in self-management and belief that CR would not improve health. Facilitators: stronger physician encouragement, improved CR education and tailored exercise programmes for older adults. |
| Graversen et al., 2017 | Denmark | Cross-sectional registry study | 5455 (3804/1651) | Women included; Low SES represented | Barriers: lack of information about CR, declining participation, low education, low income, retirement, older age, limited willingness to participate, referral to community-based rather than specialised CR. Facilitators: improved information provision, tailored referral pathways and strategies addressing socioeconomic inequalities. |
| Grewal et al., 2010 | Canada | Qualitative interpretive descriptive study | 16 (15/1) | Ethnic minority (South Asian); women included. | Barriers: language, waiting times, poor referral. Facilitators: liaison referral, multilingual information. |
| Heid & Schmelzer, 2004 | USA | Qualitative descriptive study using semi-structured interviews | 30 Females | Women included; ethnic minority participants included | Barriers: family responsibilities, transport, cost, physical limitations, work schedules and misconceptions about CR. Facilitators: physician recommendation, family support, desire to improve health and follow-up support. |
| Hutchinson et al., 2015 | New Zealand | Cross-sectional survey (hospital audit and postal questionnaire) | 143 (77/66) | Women included; ethnic minority participants included | Barriers: lack of referral, poor understanding of CR, inadequate discharge of information, older age, transport difficulties and limited culturally appropriate services. Facilitators: CR coordinator recommendation, automatic referral, improved discharge planning and culturally tailored care. |
| Hwang et al., 2017 | Australia | Mixed-methods study (surveys and semi-structured interviews) | 17 (15/2) | Women included; ethnic minority participants included; SES reported | Barriers: fear of technology, limited computer experience and technical difficulties. Facilitators: improved access to care, social support, perceived health benefits and convenience. |
| Ibsen et al., 2024 | Denmark | Mixed methods (co-production, feasibility study and process evaluation) | 260 (194/66) | Women included; low socioeconomic status; low educational attainment represented; ethnic minority participants included (linguistic/cultural barriers). | Barriers: low education, linguistic/cultural barriers, anxiety/depression, transport, work demands, comorbidity and uncertainty about CR. Facilitators: assigned contact person, systematic communication, personalised follow-up and tailored care. |
| Jin et al., 2014 | China | Cross-sectional interview study | 328 (242/86) | Women included; low educational attainment; low SES (low income and limited/no health insurance). Ethnicity not reported. | Barriers: cost, limited insurance coverage, older age, female sex, low education, low income, transport, work/time constraints. Facilitators: low-cost educational programme, flexible delivery, information on disease and risk factors. |
| Jolly et al., 2005 | UK | Cross-sectional recruitment analysis of a randomised controlled trial | 525 (402/123) | Women included; ethnic minority participants included; Low SES represented | Barriers: language barriers, inability to support minority languages, female sex and comorbidity reducing eligibility. Facilitators: Punjabi-speaking staff, bilingual recruitment and culturally appropriate trial procedures. |
| Jolly et al., 2007 | UK | Mixed methods randomised controlled trial (embedded qualitative interviews and focus groups) | 525 (398/127) | Multi-ethnic population (including South Asian participants); socioeconomically deprived populations; women included; patients with limited English proficiency | Barriers: comorbidities limiting exercise, lack of motivation (home-based participants), transport/access difficulties, communication/language barriers, poor understanding of CR, work and family commitments, parking, individual patient needs. Facilitators: home visits, telephone follow-up, Punjabi-speaking rehabilitation nurse, Punjabi Heart Manual/audio, peer support in hospital programmes, flexibility of home-based CR, comprehensive education and support. |
| Jones et al., 2007 | UK | Semi-structured interviews | 49 (33/16) | Women; ethnic minority participants included | Barriers: comorbidities, persistent cardiac symptoms, low motivation, domestic responsibilities, transport and programme accessibility. Facilitators: alternative physical activity, lifestyle changes and programme flexibility. |
| Klompstra et al., 2015 | Sweden | Cross-sectional survey | 154 (105/49) | Women included; low educational attainment represented | Barriers: low self-efficacy, lack of motivation, fatigue, family responsibilities, poor weather and fear of injury. Facilitators: higher education, greater self-efficacy and stronger exercise motivation. |
| Klompstra et al., 2021 | Sweden | Qualitative descriptive interview study | 15 (9/6) | Women included; low educational attainment reported. | Barriers: heart failure symptoms, treatment side effects, environmental barriers and uncertainty about physical activity. Facilitators: motivational support, routines, social support, perceived health benefits and adapting activities to individual abilities. |
| Klompstra et al., 2022 | Spain | Cross-sectional study | 134 (87/47) | Women included; low educational attainment represented | Barriers: low enjoyment of physical activity and increasing age. Facilitators: exercise enjoyment, higher motivation and psychological motivation for physical activity. |
| Lemstra et al., 2013 | Canada | Retrospective cohort study | 9490 | Women included; Low SES represented (low-income neighbourhoods); | Barriers: residence in low-income neighbourhoods, poor geographical access, low attendance and completion of CR. Facilitators: closer geographical access to CR facilities (although expansion alone did not significantly improve uptake) |
| Lynggaard et al., 2017 | Denmark | Randomised controlled trial | 825 (627/198) | Women included; low educational attainment; low SES represented. | Facilitators: Learning and Coping (LC) education significantly improved adherence to exercise and education sessions. Greatest benefit observed among patients with heart failure, low education and low income. |
| McGrady et al., 2009 | USA | Retrospective chart review | 380 (241/139) | Women included; ethnic minority participants included | Barriers: depression, anxiety, poor quality of life, female sex and younger age were associated with CR dropout. Facilitators: completion of CR improved exercise capacity, depression, anxiety and quality of life. |
| McSweeney & Crane, 2001 | USA | Mixed method (Descriptive naturalistic study and interview) | 40 Females | Women included; ethnic minority participants included; low educational attainment represented; low SES represented. | Barriers: lack of referral, caregiving responsibilities, transport, cost, low perceived need and comorbidities. Facilitators: physician recommendation, staff support, education, peer support and perceived health benefits. |
| Missik et al., 2001 | USA | Cross-sectional comparative study | 370 Females | Women included; educational attainment reported | Barriers: lack of physician referral, inadequate insurance coverage, limited transportation and accessibility. Facilitators: physician referral, insurance coverage, CABG, higher education and available transportation increased participation |
| Mochari et al., 2006 | USA | Cross-sectional study | 304 Females | Women included; ethnic minority participants included; educational attainment reported; SES reported. | Barriers: low physician referral among minority women, financial barriers. Facilitators: physician referral/instruction to attend CR. |
| Nakayama et al., 2020 | Japan | Prospective cohort study | 9019 (5543/3476) | Women included; Rural/remote populations (geographical access) | Barriers: longer road distance/travel time, low albumin, heart failure, valvular and aortic disease. Facilitators: shorter travel distance, PCI/CABG. |
| Nalini et al., 2014 | Iran | Observational study | 4735 (3395/1340) | Women included; rural/remote populations (geographical access); low SES reported | **Barriers:** lower referral and participation among women and patients living outside Kermanshah. **Facilitators:** systematic referral and hybrid CR programme improved participation, particularly for women and rural patients. |
| Pedersen et al., 2018 | Denmark | Prospective observational study | 302 (218/84) | Women included; Low educational attainment | Barriers: low education, comorbidity, living alone, long commute. Facilitators: higher self-efficacy, shorter commute. |
| Plach et al., 2002 | USA | Cross-sectional descriptive study | 157 Females | Women included; ethnic minority participants included; low educational attainment represented; low SES represented. | Barriers: lack of referral, health problems, time constraints, inconvenience (distance), personal preference. Facilitators: physician referral and perceived importance of CR. |
| Poh et al., 2015 | Singapore | Prospective observational study | 418 (376/42) | Women included; ethnic minority participants included | Barriers: busy work schedules, preference for self-exercise, lack of perceived need and restrictive eligibility criteria. Facilitators: revised referral criteria and supervised CR. |
| Pozehl et al., 2018 | USA | Randomised controlled trial | 204 (113/91) | Women included; ethnic minority participants included | Barriers: poor long-term exercise adherence. Facilitators: HEART Camp intervention, coaching, education and social support improved exercise adherence. |
| Resurrección et al., 2018 | Spain | Qualitative study using semi-structured interviews | 10 Females | Women included; low educational attainment represented | Barriers: poor physical and mental health, caregiving and work responsibilities, transport and programme logistics, long waiting times. Facilitators: flexible/modular CR, psychological support and community-based services. |
| Rodrigo et al., 2021 | Netherlands | Retrospective observational cohort study | 469 (341/128) | Women included; low SES represented. | Barriers: older age, greater distance to CR centre, previous CVD, NSTEMI/unstable angina. Facilitators: systematic referral and shorter travel distance improved participation. |
| Rogerson et al., 2012 | Australia | Qualitative study using in-depth semi-structured interviews | 15 (9/6) | Women included; Low educational attainment represented; SES reported. | Barriers: low mood, low motivation, fear of exercise, limited knowledge and physical limitations. Facilitators: social support, psychological strategies, perceived benefits and having a purpose for exercise. |
| Rouleau et al., 2018 | Canada | Qualitative study using face-to-face semi-structured interviews | 14 (11/3) | Women included; ethnic minority participants included; Low educational attainment represented; SES reported. | Barriers: travel, programme costs, scheduling conflicts, uncertainty about ability to attend and psychological distress. Facilitators: healthcare provider recommendation, perceived benefits, family support and positive first impressions of CR. |
| Sánchez-Delgado et al., 2016 | Colombia | Cross-sectional study | 30 (20/10) | Women included; low educational attainment represented; low SES represented. | Barriers: exercise perceived as tiring or painful, lack of awareness of CR, perceived no need because already exercising, costs, comorbidities/functional limitations, perceived needs. Facilitators: greater education regarding CR benefits and referral to CR. |
| Sanderson et al., 2003 | USA | Retrospective cohort study | 526 (341/185) | Women included; ethnic minority participants included | Barriers: employment, obesity, smoking, poor functional capacity, high clinical risk, medical complications. Facilitators: better functional capacity and interventions targeting patients with low exercise capacity to improve programme completion. |
| Sangster et al., 2013 | Australia | Cross-sectional comparative study | 313 (228/85) | Women included; low SES represented; low educational attainment represented. | Barriers: greater distance from CR services, poorer access for rural residents, obesity, physical inactivity, lower confidence and social support, lower attendance at outpatient CR. Facilitators: telephone-based CR, innovative remote delivery models, living closer to CR services. |
| Savage et al., 2013 | Canada | Qualitative interviews study | 20 Males | Low socioeconomic status (underprivileged men); low educational attainment represented. | Barriers: financial hardship, fatalistic beliefs, short-term priorities, physical limitations and limited social support. Facilitators: None specifically identified (study primarily explored barriers). |
| Schopfer et al., 2020 | USA | Mixed methods study (survey and semi-structured interviews) | Survey: 171 (170/1); Qualitative: 10 (10/0) | Ethnic minority participants included. | Barriers: poor understanding of CR, competing priorities, low motivation, inadequate consultation and financial concerns. Facilitators: improved education, motivational interviewing and repeated follow-up. |
| Sérvio et al., 2019 | Brazil | Cross-sectional study | 805 (489/316) | Women included; educational attainment reported; Low SES represented. | Barriers: lack of CR resources and programmes, poor referral, comorbidities/functional limitations, perceived need, personal/family issues, access barriers (distance, transport, cost), travel/work conflicts. Facilitators: improved funding, increased programme availability, systematic referral and advocacy for CR services. |
| Shanmugasegaram et al., 2013 | Canada | Cross-sectional study | 1809 (1357/452) | Women included; ethnic minority participants included; low educational attainment represented; low SES represented. | Barriers: distance, transportation, cost, lower referral, lower enrolment and fewer CR sessions. Facilitators: CABG associated with lower perceived barriers; strategies to improve equitable access to CR. |
| Snoek et al., 2021 | Netherlands, Denmark, Spain, France & Switzerland (5 European countries) | Multicentre randomised controlled trial | 179 (145/34) | Women included; ethnic minority participants included | Barriers: refusal of centre-based CR, transportation difficulties, perceived lack of usefulness of CR. Facilitators: home-based mobile CR with telemonitoring and motivational interviewing significantly improved exercise capacity and provided an effective alternative for non-participants. |
| Soroush et al., 2018 | Iran | Cross-sectional study | 312 (199/113) | Women included; educational attainment reported; Low SES represented. | Barriers: greater distance to CR centre, unemployment/non-official employment, physiological illness perceptions, low referral rate (8.3%). Facilitators: official employment, previous coronary history, hyperlipidaemia, improved awareness of CR benefits and improved access to CR centres. |
| Stiller et al., 2004 | USA | Observational (retrospective cohort with patient survey) | 203 (148/55) | Women included; ethnic minority participants included; SES reported. | Barriers: low referral overall, transportation, distance, work conflicts, domestic responsibilities, insurance, age and physician-related factors explored. Facilitator: male physician gender was the only independent predictor of referral in multivariable analysis. |
| Sutantri et al., 2019 | Indonesia | Exploratory qualitative study using semi-structured interviews | 23 Females | Women included; different Indonesian ethnic groups; low SES reported. | Barriers: family and social role expectations. Facilitators: healthcare staff recommendation, family support, peer support and health insurance. |
| Tadaki et al., 2020 | Japan | Multicentre prospective cohort study | 4935 (3503/1432) | Women included; SES reported. | Barriers: disease-related limitations (particularly non-orthopaedic conditions), busyness, lack of exercise habits, dislike of exercise and socioeconomic reasons. Facilitators: perceived adequate daily physical activity ("busyness") was associated with better prognosis. |
| Thompson et al., 2024 | USA | Retrospective cohort study | 143392 (80040/62952) | Women included; ethnic minority participants included; Low SES represented. | Barriers: low overall CR uptake after heart failure, admission to non-advanced HF centres. Facilitators: admission to advanced heart failure centres increased CR participation and earlier CR initiation. |
| Tunsi et al., 2023 | Saudi Arabia | Exploratory qualitative study using semi-structured interviews | 21 (13/8) | Women included; low educational attainment represented (most participants had secondary education or below); Saudi population (100% Arab); SES reported | Barriers: sociocultural norms, inadequate healthcare services, environmental and policy barriers. Facilitators: family support, religious beliefs and sociocultural values. |
| Turk-Adawi et al., 2014 | USA | Multicentre observational study | 6874 (4763/2111) | Women included; ethnic minority participants included; educational attainment reported; SES reported. | Barriers: older age, heart failure, diabetes, depression, smoking and lack of insurance. Facilitators: health insurance, CABG, CR programme promotion, AACVPR certification and rural programme location improved enrolment. |
| Vonk et al., 2021 | Netherlands & Germany | Multicentre cross-sectional survey | 1829 (1265/564) | Women included; low educational attainment represented. | Barriers: no need for CR or supervision, exercising, physical problems and lack of programme personalisation. Facilitators: better patient information, motivation and more personalised/home-based CR. |
| Wang et al., 2024 | China | Qualitative study using semi-structured interviews | 40 (28/12) | Women included; low educational attainment represented; SES reported; Chinese population | Barriers: fragmented CR services, difficulty integrating CR into daily life and unmet rehabilitation needs. Facilitators: collaborative care, personalised rehabilitation plans, trust in healthcare professionals and positive outcome expectations. |
| Warehime et al., 2020 | USA | Qualitative multiple-case study using semi-structured interviews (directed content analysis) | 22 (12/10) | Women included; ethnic minority participants included (Black) | Barriers: health complications, lack of routine, low motivation and limited time. Facilitators: perceived health improvements, social support, exercise knowledge, motivation/perseverance and establishing an exercise routine. |
| Winnige et al., 2021 | Czech Republic | Prospective observational validation study | 186 (144/42) | Women included; low educational attainment represented; SES reported. | Barriers: distance, work responsibilities, lack of time, transportation, comorbidities, travel, COVID-19 restrictions. Facilitators: fewer barriers among CR completers; education and geographical accessibility associated with participation. |
| Wittmer et al., 2011 | Switzerland | Prospective cohort study | 2371 (2015/356) | Women included; low educational attainment represented | Barriers: smoking, diabetes, obesity, low exercise capacity, widowhood, lower education. Facilitators: higher exercise capacity, lower BMI, non-smoking status and better quality of life predicted programme completion. |
| Wong et al., 2016 | Hong Kong | Qualitative descriptive study using in-depth interviews | 22 (19/3) | Women included; low educational attainment represented; SES reported | Barriers: work commitments and maintaining exercise after programme completion. Facilitators: positive attitudes, peer support, family/workplace support and culturally appropriate exercise. |
| Yang et al., 2023 | China | Qualitative descriptive study using semi-structured interviews | 16 (9/7) | Women included; low educational attainment represented | Barriers: lack of knowledge, activity intolerance, fear of exercise, role conflict and low self-efficacy. Facilitators: tailored education, psychological support and personalised rehabilitation strategies. |
| Yang et al., 2023 | China | Cross-sectional study | 215 (103/ 112) | Women included; low educational attainment represented; Low SES represented | Barriers: fear of movement (kinesiophobia), older age. Facilitators: higher education, greater social support and exercise self-efficacy improved home-based CR adherence. |
| Yates et al., 2003 | USA | Cross-sectional comparative study | 222 (163/58) | Women included; ethnic minority participants included; educational attainment reported; SES reported. | Barriers: lack of physician explanation/referral, greater distance from CR, reimbursement issues, comorbid illness. Facilitators: physician recommendation, information during hospitalisation and living close to a CR programme increased participation. |
| Zhang et al., 2017 | USA | Retrospective cohort study | 590 (359/231) | Women included; ethnic minority participants included; Low SES represented | Barriers: non-White ethnicity, lack of insurance, copayments and younger age (for adherence). Facilitators: insurance coverage and absence of copayments improved initiation and adherence. |

Behaviour

Capability

Physical capability

TDF: Skills

Psychological capability

TDF: Knowledge

TDF: Memory, attention and decision processes

TDF: Behavioral Regulation

Opportunity

Physical Opportunity

TDF: Environmental Context and Resources

Social Opportunity

TDF: Social Influences

Motivation

TDF: Beliefs about Capabilities

TDF: Beliefs about Consequences

TDF: Intentions and goals

TDF: Social/Professional Role and Identity

Automatic Motivation

Emotion

**Supplementary Figure 1:** Capability-Opportunity Motivation-Behaviour (COM-B) and Theoretical Domains Framework (TDF) Behaviour Change Domains.

**Identification**

Studies included in review (n = 85)

Qualitative studies (n = 22)

Mixed Method studies (n = 9)

Quantitative studies (n = 54)

Full-text articles excluded (n = 65)

wrong outcome (n = 25)

wrong intervention (n = 2)

wrong population (n = 34)

wrong publication type (n = 4)

Full-text articles assessed for eligibility

(n = 150)

Records excluded

(n = 21,211)

Records screened

(n = 21,361)

Records removed *before screening*:

Duplicate records removed

(n = 7,432)

Records identified through databases

MEDLINE, PsycINFO, CINAHL, PubMed, and Scopus

(n = 28,793)

**Included**

**Eligibility**

**Screening**

**Supplementary Figure 2:** PRISMA flow diagram of databases and citation searching for the identification, screening, eligibility and inclusion of studies.
